# Concerted and Stepwise Proton-Coupled Electron Transfer for Tryptophan-Derivative Oxidation with Water as the Primary Proton Acceptor: Clarifying a Controversy

**DOI:** 10.1021/jacs.2c00371

**Published:** 2022-04-13

**Authors:** Astrid Nilsen-Moe, Andrea Rosichini, Starla D. Glover, Leif Hammarström

**Affiliations:** Department of Chemistry, Ångström Laboratory, Uppsala University, P.O. Box 523, 75120 Uppsala, Sweden

## Abstract

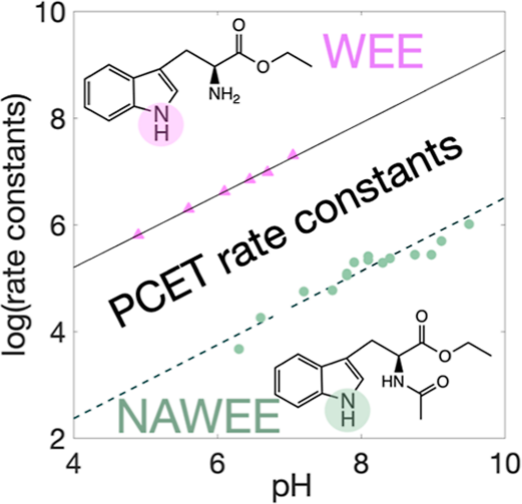

Concerted electron-proton transfer (CEPT) reactions avoid charged intermediates and may be energetically favorable for redox and radical-transfer reactions in natural and synthetic systems. Tryptophan (W) often partakes in radical-transfer chains in nature but has been proposed to only undergo sequential electron transfer followed by proton transfer when water is the primary proton acceptor. Nevertheless, our group has shown that oxidation of freely solvated tyrosine and W often exhibit weakly pH-dependent proton-coupled electron transfer (PCET) rate constants with moderate kinetic isotope effects (KIE ≈ 2–5), which could be associated with a CEPT mechanism. These results and conclusions have been questioned. Here, we present PCET rate constants for W derivatives with oxidized Ru- and Zn-porphyrin photosensitizers, extracted from laser flash-quench studies. Alternative quenching/photo-oxidation methods were used to avoid complications of previous studies, and both the amine and carboxylic acid groups of W were protected to make the indole the only deprotonable group. With a suitably tuned oxidant strength, we found an ET-limited reaction at pH < 4 and weakly pH-dependent rates at pH > ∼5 that are intrinsic to the PCET of the indole group with water (H_2_O) as the proton acceptor. The observed rate constants are up to more than 100 times higher than those measured for initial electron transfer, excluding the electron-first mechanism. Instead, the reaction can be attributed to CEPT. These conclusions are important for our view of CEPT in water and of PCET-mediated radical reactions with solvent-exposed tryptophan in natural systems.

## 1 Introduction

Tryptophan (W) is one of only a handful of amino acids that can undergo proton-coupled electron transfer (PCET). In proteins, W partakes in PCET-mediated pathways that transfer electrons between different redox-active amino acids up to more than 10 Å apart to reach its destination. While electron transfer can be long-range, the coupled protons are transferred over much shorter distances (<1 Å) in each step to either a neighboring amino acid or water.^1−5^ Nevertheless, this leads to net transport of radicals over large distances to and from specific redox sites. There is consequently great interest in tryptophan PCET reactions in both proteins and smaller molecular model systems.^6−10^

**Figure 1:**
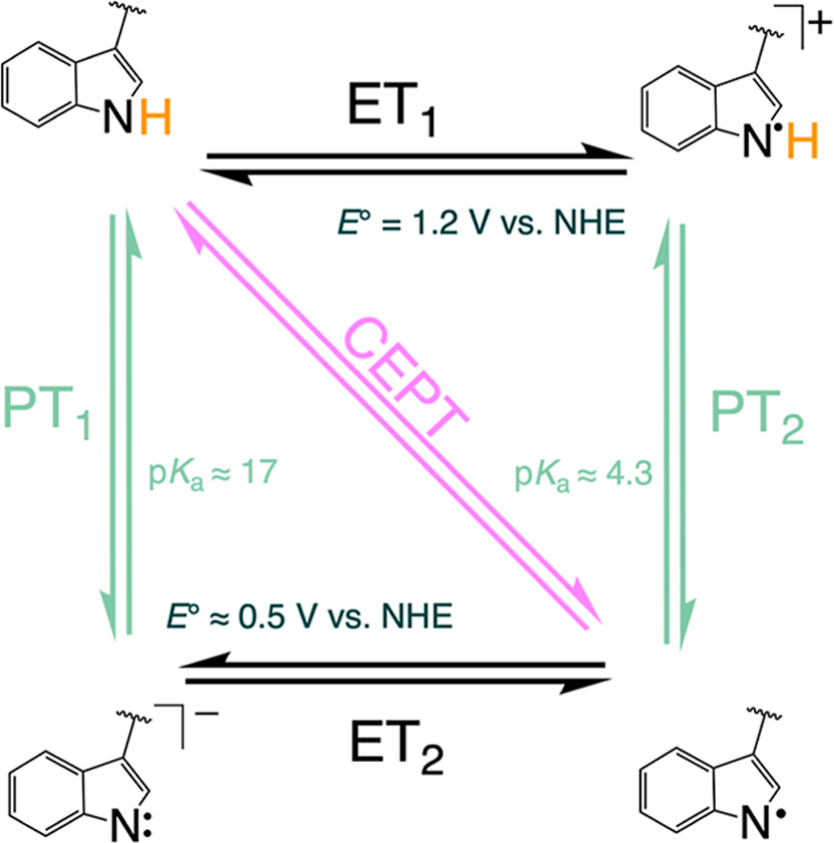
Mechanisms for PCET in tryptophan (W) with an implied external oxidant and base. The transferred proton is marked in orange. The radical is schematically shown to reside on the indole nitrogen; however, electron spin resonance shows that the unpaired spin density is delocalized.^15^ Black horizontal arrows represent ET, green vertical arrows represent PT, and pink diagonal arrows represent CEPT.

Figure 1 shows the different PCET pathways using W as an example. PCET may occur in two consecutive steps, shown with black and green arrows. Here, either initial electron transfer (ET_1_) is followed by proton transfer from the radical cation (PT_2_) or initial proton transfer (PT_1_) is followed by electron transfer from the base form (ET_2_). PCET may also occur in one concerted step, shown with a diagonal pink arrow. In a stepwise reaction, the first step often has only a small driving force or is even uphill, which tends to make the reaction slow. In hydrophobic environments in particular, such as the interior of many proteins, the charged intermediates are not favored.^11^ The concerted electron–proton transfer (CEPT) reaction avoids charge buildup, and the driving force is typically more favorable as it equals the sum of those for the involved electron-transfer (ET) and proton-transfer (PT) steps. CEPT reactions therefore tend to have a lower activation barrier relative to the stepwise mechanisms. On the other hand, non-adiabatic CEPT is limited by a lower probability of two particles tunneling, which may result in a smaller kinetic pre-factor. The balance between these factors can explain the competition between the sequential and concerted reactions.^12,13^ In general, a stronger oxidant tends to favor sequential ET followed by PT (ETPT), while a stronger proton accepting base favors PT followed by ET (PTET). When both the oxidant and base are weak, CEPT can instead be the favored pathway. Thus, the mechanism can be varied by changing the driving force for PT and/or ET. The free-energy dependence of the rate constant can also be used to determine which mechanism dominates for a given range of conditions. A supporting piece of information when determining the PCET mechanism is the kinetic isotope effect (KIE), where the transferring proton is substituted for a deuteron. KIEs can be used to gauge the effect of proton transfer on the observed rate constants. One should note that there are many factors that may influence the KIE; as such, it is not sufficient evidence to fully establish a particular mechanism.^12,14^

W often undergoes stepwise ETPT and sometimes pure ET reactions in proteins. This can be rationalized by its relatively low redox potential (*E*°(W^•+^/W) ≈ +1.21 V vs NHE)^16^ and low acidity of the indole proton (Figure 1) in the reduced state (p*K*_a_(W) = ∼17, p*K*_a_(W^•+^) = ∼4.3).^17,18^ In a protein, only the indole group of W may react via PCET since the other functional groups make up the peptide bond. Henceforth, the p*K*_a_ of W and its analogues refers to the indole group. Unless otherwise stated, W is assumed to be protonated on the amine side group below pH 7.5 and deprotonated above this pH. The carboxylic group p*K*_a_ ≈ 2.5, but this group is converted to an ester in WEE and NAWEE used in the present study. The large p*K*_a_ of W means that sequential PTET can in many instances be ignored. When water acts as the primary proton acceptor, CEPT reactions are less favored than the primary ET step of ETPT; the low p*K*_a_ value of protonated water (H_3_O^+^_(aq)_, p*K*_a_ ≡ 0) makes it thermodynamically unfavorable to transfer a proton from W^•+^ to a small cluster of water molecules. Following this reasonable argument,^19^ it has been proposed that CEPT from tryptophan is not likely to compete with ETPT if water (H_2_O) is the primary proton acceptor because of a smaller driving force than for pure ET and a smaller probability for electron and proton tunneling.^7,20,21^ Nonetheless, both covalently linked Ru(III)polypyridine-tryptophan (Ru(III)-W) and bimolecular systems studied by our group have directly or indirectly demonstrated that CEPT from tryptophan with water as the proton acceptor is viable.^6,9,22,23^ In both cases, control experiments excluded OH^–^ and buffer species as primary proton acceptors. It was found that ETPT dominated the reaction when *E*°(Ru(III/II)) ≳ *E*°(W^•+^/W). With lower oxidant potentials, the reaction followed a CEPT mechanism. When ETPT was operative, the KIE was ≈1 and rate constants were pH-independent. CEPT for the Ru(III)-W dyads was characterized by distinct KIE ≈ 3.5 (at neutral pH) and rate constants that increased weakly with pH, ca. 3-fold per pH unit.^19^ This is much weaker than the 10-fold increase expected for a stepwise reaction via the W^–^ anion or for a CEPT reaction with OH^–^ or base forms of the buffer. The theoretical origin of this pH dependence remains unclear.

**Figure 2:**
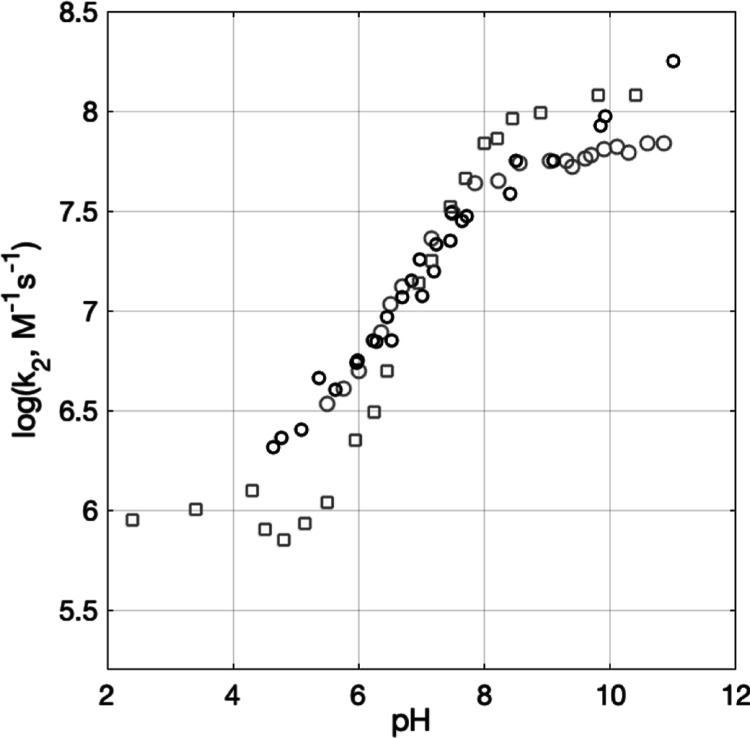
Previously published second-order rate constants for WEE oxidation by [Ru(dmb)_3_]^3+^ as a function of pH from ref (22) (shown in black) and ref (7) (shown in gray). Flash-quench photolysis of [Ru(dmb)_3_]^2+^ with MV^2+^ (circles) or [Ru(NH_3_)_6_]^3+^ (squares) as the quencher was used to produce [Ru(dmb)_3_]^3+^.

**Scheme 1:**
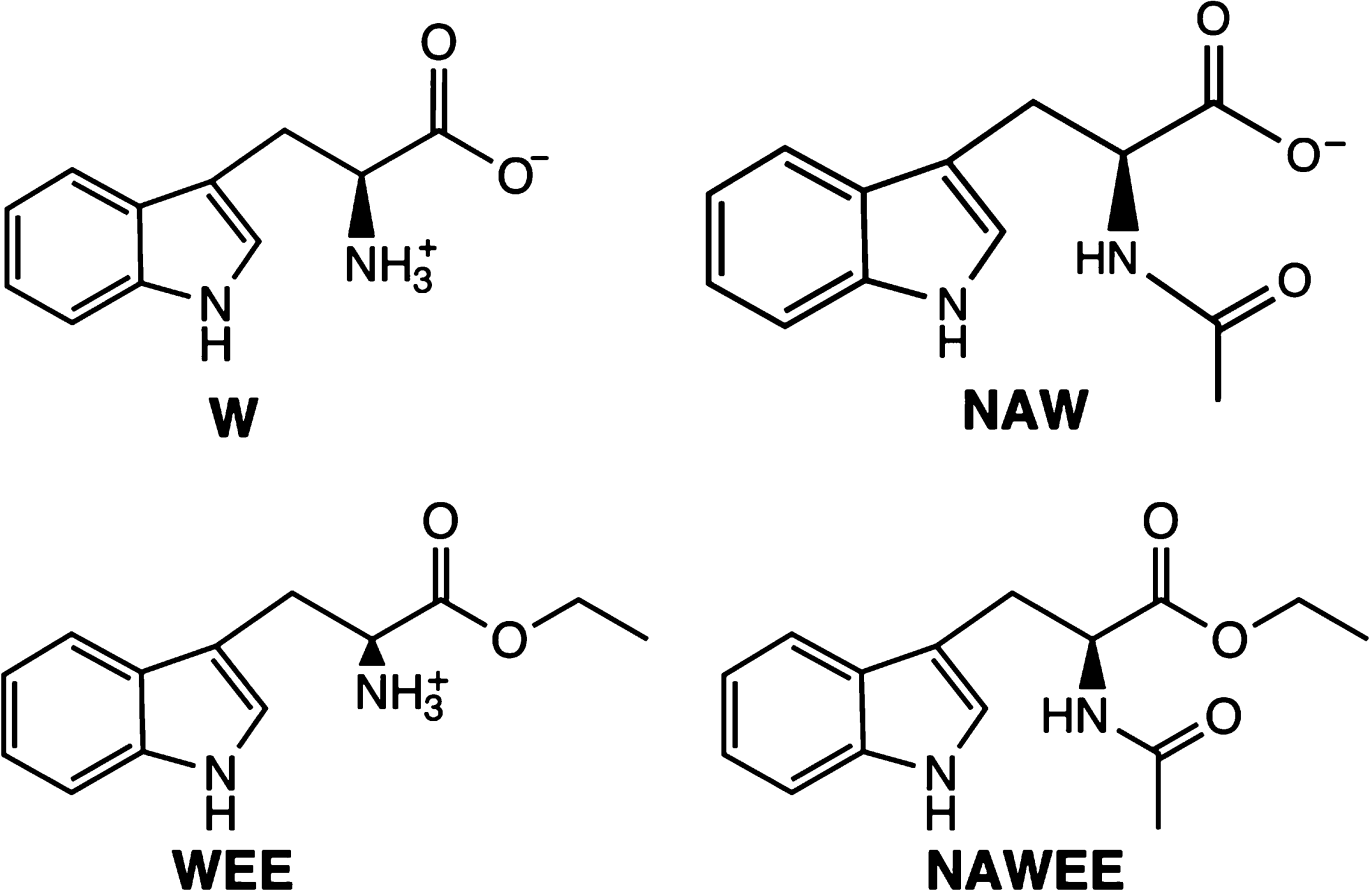
Chemical Structures of Tryptophan and Its Three Analogues Discussed in This Paper at pH = 7

In a study of bimolecular PCET,^22^ rate constants were determined for two W analogues, WEE and NAW (Scheme 1), using flash-quench-generated [Ru(dmb)_3_]^3+^ (dmb = 4,4′-dimethyl, 2,2′-bipyridine) as the external oxidant and transient spectroscopy to determine reaction kinetics. Analogues of W have protected carboxylic and/or amine groups, as was the case in Ru(III)-W, with the purpose of avoiding interference from PT that is not from the indole proton. Significant KIEs of ca. 2.5 and pH-dependent rate constants were observed for WEE (Figure 2, black circles). These data supported a CEPT mechanism in WEE when a weak oxidant, [Ru(dmb)_3_]^3+^ (Table 1), is used.^22^ This showed that the weakly pH-dependent rate constants and significant KIEs were more general and not exclusive to the particular Ru(III)-W dyad structure used in the previous studies. The assignment of a concerted mechanism in WEE was challenged in a follow-up study by Bonin et al.^7^ The alternate conclusions from these authors are summarized in three points below:

**Table 1:** Names, Abbreviations, and Apparent Reduction Potentials for Tryptophan, Three Analogues, and Photosensitizers Used

name (abbreviation)	*E*° vs NHE (V) (W^•^H^+^/W)	*E*°′ vs NHE (V) at pH 5.2 (W^•^/W)
tryptophan (W)	1.21^16^	1.15^a^
*n*-acetyl tryptophan (NAW)	∼1.1^8^	1.0^a^
tryptophan ethyl ester (WEE)		1.00^b^
*n*-acetyl tryptophan ethyl ester (NAWEE)		0.908^b^

name	abbreviation, redox couple	*E*° vs NHE (V)
ruthenium(II) 4,4′-dimethyl-2,2′-bipyridine	[Ru(dmb)_3_]^3+/2+^	1.10^26^
zinc(II) meso-5,10,15,20-tetrakis (4-sulphonato phenyl) porphyrine	[ZnTPPS]^3–/4–^	0.87^27^

a Calculated from *E*° using p*K*_a_(W^•^H^+^) = 4.3.^17,18^

b Measured using the Ag/AgCl reference electrode and recalculated to NHE using *E*°(Ag/AgCl) = +0.205 V versus NHE.^28^

Point 1. PCET rate constants for WEE were suggested to be limited by ET in the entire pH range studied (Figure 2, gray symbols), meaning that the mechanism was either ET (at pH < 4.5) or ETPT. Since ET-limited reactions should exhibit no pH dependence, it was suggested that the observed pH dependence arose from changes in Δ*G*°_ET_ due to the electrostatic work terms of the encounter–successor complex. The change in electrostatics with pH was suggested to originate from the −NH_2_ group on WEE which has a p*K*_a_ of 7.5.^7^ The p*K*_a_ of the NH_2_/NH_3_^+^ side group shifts to 8 in D_2_O, which was used to explain the previously reported KIE at neutral pH. The work terms were calculated to give a 130 mV difference between the high and low pH and would explain the rate constants that span 2 orders of magnitude. Note that PT to the NH_2_/NH_3_^+^ side group could be excluded by the fact that the rate was first-order in [WEE].^7^

Point 2. It was suggested^7^ that the rate constants of WEE oxidation determined in ref (22) (black circles in Figure 2) were not reliable because the oxidative quencher used in the flash-quench experiments, methyl viologen (MV^2+^), forms an adduct with WEE, in particular at higher pH values. The rate constants for WEE oxidation were therefore redetermined using [Ru(NH_3_)_6_]^3+^ as the oxidative quencher (gray squares, Figure 2). The disagreement in rate constants below pH 6 when MV^2+^ or [Ru(NH_3_)_6_]^3+^ was used as the quencher was explained by uncertainties caused by competitive charge recombination between oxidized [Ru(dmb)_3_]^3+^ and the reduced quencher. At lower pH values, WEE oxidation rates are slower, resulting in observed kinetics that are increasingly dominated by the competitive recombination reaction.

Point 3. The oxidation of NAWEE was investigated in ref (7). In NAWEE, both the carboxylic acid and amine groups are protected, leaving the indole proton as the only titratable group on the molecule (Scheme 1). From kinetic data, the rate constants of NAWEE oxidation by [Ru(dmb)_3_]^3+^ were found to be pH-independent. From electrochemical data, two irreversible cyclic voltammograms were used to compare NAWEE and WEE oxidation. NAWEE was proposed to be “less oxidizable” than WEE by ∼50 mV. The 3-fold slower reaction for NAWEE than WEE at high pH was taken as consistent with an ET-limited ETPT reaction for both compounds. It was thus concluded that the observed pH dependence of the rate constant for WEE was only due to titration of the NH_2_/NH_3_^+^ side group (p*K*_a_ ∼ 7.5).

In this report, we re-evaluate PCET in WEE and NAWEE. We show how titration of the NH_2_/NH_3_^+^ side group cannot explain the pH-dependent rate constant for WEE using (i) analyses of data pertaining to electrostatic effects, where we show that the explanation of the pH-dependent rate constant in ref (7) is based on an incorrect evaluation of work terms; (ii) voltammetry to determine the apparent W^•^/W potentials of WEE and NAWEE, which show that NAWEE is oxidized at a lower potential than WEE, opposite to what was stated in ref (7); (iii) determination of rate constants for WEE oxidation by [Ru(dmb)_3_]^3+^ as a function of pH; and (iv) determination of rate constants for NAWEE oxidation by a weaker oxidant (zinc(II)tetra(4-sulphonatophenyl)porphyrin, [ZnTPPS]^4–^) as a function of pH. In flash-quench experiments, we remove any kinetic uncertainties due to competitive recombination by using an irreversible oxidative quencher. The new data clearly demonstrate that the PCET reaction is not limited by ET over the entire pH range for both WEE and NAWEE when appropriate oxidants are used. PCET kinetics for NAWEE, with both side groups protected, show a pH dependence of of the PCET rate constants that is parallel to that for WEE, provided that the oxidant strength is decreased to allow for a CEPT mechanism to compete. Our results show a general behavior of tryptophan PCET reaction in aqueous solution, which contrasts with previous theoretical predictions, and we discuss the implications thereof.

## 2 Results and Discussion

In this report, we re-examine the PCET reactivity of the tryptophan derivatives WEE and NAWEE in aqueous environments. In Section 2.1, we describe the electrostatic work-term calculation which was incorrectly applied in ref (7). Section 2.2 describes voltammetry experiments to determine the apparent W^•^/W potentials for WEE and NAWEE to establish how protecting the amine group affects the indole reduction potential. Section 2.4 focuses on rate constants for WEE oxidation at pH 2.0–11.4 by transient absorption spectroscopy with laser flash-quench photolysis to generate the oxidant in situ. The use of an irreversible quencher or direct two-photon ionization without a quencher simplified analyses of the observed kinetics by avoiding potential effects from complexation with MV^2+^ or the instability of [Ru(NH_3_)_6_]^3+^ at pH ≥ 8. In Section 2.5, we report pH-dependent rate constants for NAWEE oxidation using a weak oxidant between pH 6.3 and 9.5.

### 2.1 Electrostatics of the Encounter/Successor Complex Cannot Explain pH-dependent Rate Constants for WEE

Here, we consider the pH-dependent oxidation of WEE by an external diffusing oxidant, [Ru(dmb)_3_]^3+^. Bonin et al.^7^ argued that the pH dependence of WEE oxidation arose from changes in the electrostatic interactions between reactants and products as the −NH_2_/–NH_3_^+^ equilibrium (p*K*_a_ = 7.5) shifted with pH (Point 1, Introduction). Specifically, the Coulombic work needed to bring the ET reactants and products to the reaction distance in solution is not the same for the −NH_2_ and −NH_3_^+^ forms. For simplicity of notation, we use WEE throughout the present paper to indicate both these protonation forms and use the charge in WEE^•+^ to specifically indicate the protonated indole radical.

The Coulombic work is defined as *w* = *z*_1_*z*_2_*e*^2^/4πε_0_ε_S_*d*, where *z*_1_ and *z*_2_ are the ion charge numbers, *e* is the elementary charge, ε_0_ is the electric constant (permittivity), ε_S_ is the solvent static dielectric constant, and *d* is the distance between ions. The Coulombic work for reactant and product states contributes to Δ*G*°_ET_ in the encounter complex such that^24^
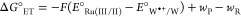
1where *w*_R_ and *w*_P_ are the Coulombic work terms associated with the reactants and products, respectively. The work terms for acid and base forms of WEE (*w*_A_ and *w*_B_, respectively) will be not be the same, and a shift in Δ*G*°_ET_ can be expected.

It was concluded in ref (7) that WEE oxidation by [Ru(dmb)_3_]^3+^ occurred by ET-limited ETPT over the entire pH range examined. The pH-dependent rate constants reported in ref (7) (Figure 2, gray squares) were fit to a model of titration of the −NH_2_/–NH_3_^+^ group of WEE. In this model, the driving force was assumed to be more favorable for the base form due to the difference between work terms in the acid (−NH_3_^+^) and base (−NH_2_) forms of WEE. That is, Δ*G*° for ET was calculated to be 130 meV more negative for the base form of WEE. With this value, the model in ref (7) could fit the pH-dependent data, giving a 100-fold higher ET rate constant for the base form.

The work terms were incorrectly calculated in ref (7), which led to an erroneous conclusion. The work term effect on Δ*G*°_ET_ was calculated by taking the sum of the reactant and product work terms; for a correct thermodynamic cycle, it must be the difference between reactant and product work terms.^24^ We rectify this calculation below and show that this gives instead an opposite and quite small effect on Δ*G*°_ET_.

At pH < 7.5, the reactants in the bimolecular encounter–successor complex for ET are [Ru(dmb)_3_]^3+^ and the monocationic WEE, where WEE is protonated at the amine. The products are [Ru(dmb)_3_]^2+^ and the dicationic radical (NH_3_^+^-WEE^•+^), where both the indole radical and amine groups remain protonated. The work terms are given by eq 2a. At pH > 7.5, the reactants are [Ru(dmb)_3_]^3+^ and the charge-neutral WEE; upon ET, the products are [Ru(dmb)_3_]^2+^ and WEE^•+^, where the latter is only protonated at the indole radical and the work terms are described by eq 2b
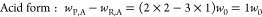
2a
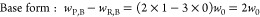
2b

In eqs 2a and 2b, *w*_0_ = *e*_0_^2^/4πε_0_ε_S_*d*. Using *w*_0_ = +26 meV as estimated by Bonin et al.,^7^ the work term contributions to Δ*G*°_ET_ should be +26 and +52 meV for the acid and base forms, respectively. Bonin et al. incorrectly calculated the acid work term to be 7*w*_0_ = +180 meV, which led to an overestimation between the differences in ET driving force between acid and base forms of WEE. The work terms are instead very small, and the base form actually shows the more positive Δ*G*°, contrary to what was reported previously. Consequently, the pH-dependent data in Figure 2, with a rate constant that increases with pH, cannot be explained by an ET-limited reaction with a driving force that is electrostatically modulated by the protonation of the amine group.

The change in electrostatics with pH due to the protonation equilibrium of the ammonium group on WEE may still influence the diffusional encounter rate of the reacting species. To determine how much this might affect the rate constants, we measured transient absorption (vide infra) with 200 mM KCl added to the solution at three different pH values. The results are seen in Figure S5 and show rate constants that are identical to those for samples without KCl, within experimental uncertainty. If electrostatic effects on the diffusional encounter between WEE and [Ru(dmb)_3_]^3+^ had been important, we would have expected to see a significant screening effect by 200 mM KCl. Electrostatic differences between the −NH_2_/–NH_3_^+^ forms of WEE are therefore not the origin of the changes in rate constant, which is 2 orders of magnitude higher at pH > 8 than at pH < 4. It means that it is difficult to explain the data in Figure 2 with an ET-limited oxidation of WEE in the entire pH range, as was proposed in ref (7).

### 2.2 Apparent W^•^/W Reduction Potentials Show that NAWEE is Easier to Oxidize than WEE

The model of ET-limited ETPT in WEE proposed by Bonin et al.^7^ was further supported by comparing the pH dependence of PCET rate constants in a related tryptophan analogue, NAWEE, Scheme 1. When the same [Ru(dmb)_3_]^3+^ oxidant was used, NAWEE exhibited pH-independent oxidation rate constants, which was interpreted as an ET-limited process in ref (7). By comparing two ill-defined cyclic voltammograms at pH 10, it was proposed that NAWEE was more difficult to oxidize than WEE. If NAWEE is more difficult to oxidize and still exhibits rate constants consistent with an ET-limited ETPT process, then one can assume that WEE oxidation must also be ET-limited ETPT as ETPT should be disfavored relative to CEPT when the driving force for ET is decreased.^12^ That is, if the reduction potential for NAWEE is higher than that for WEE, the reaction mechanism should not change due to differences in ET driving force between WEE and NAWEE.

Functionalizing the amine group on W to form NAW has previously been shown to shift the reduction potential by 100 mV to lower values, see Table 1.^8^ The shifts in reduction potentials between WEE and NAWEE reported by Bonin et al. were opposite to what was observed for W and NAW; this counter-intuitive observation led us to reinvestigate the apparent reduction potentials for WEE and NAWEE.

We observed similarly ill-defined voltammograms at high pH to those previously reported;^7^ however, at pH 5.2, we obtained well-defined anodic peaks. Using cyclic voltammetry, peak potentials were determined as a function of scan rate for WEE and NAWEE at pH 5.2 in 0.1 M KNO_3_ and 0.5 mM KP_i_. From the intercept of peak potentials versus scan rate (slope ∼20 mV per decade), the apparent W^•^/W potentials for the two compounds were determined (Table 1, details on page S3 in the Supporting Information). Our data show that when the amine group is protected, the potential shifts by ∼100 mV to lower values for NAWEE compared to WEE. This is the opposite trend to what was concluded previously;^7^ therefore, the pH independence of NAWEE cannot be used to disprove a CEPT mechanism for WEE. The difference in pH dependence could instead be due to different PCET mechanisms, and we show below that this is most likely the case.

We note that deprotonation of the −NH_3_^+^ group does not give a detectable kink in the Nernstian slope of Pourbaix diagrams for W;^25^ therefore, changes in *E*° cannot explain the 100-fold larger rate constant for the base form of WEE in the framework of an ET-limited mechanism.

### 2.3 Oxidation of W Analogues by Flash Photolysis

Figure 2 summarizes the second-order rate constant of WEE oxidation by [Ru(dmb)_3_]^3+^ from two different studies. The rates of WEE oxidation change with pH and show some discrepancies at high- and low-pH regions depending on the oxidative quencher used.^7^ In this study, we carefully reinvestigated the pH dependence of WEE oxidation using two different methods to obtain laser-flash-generated [Ru(dmb)_3_]^3+^. The rate constants of WEE oxidation were determined by following the oxidation reaction using optical transient absorption (TA) spectroscopy.

**Figure 3:**
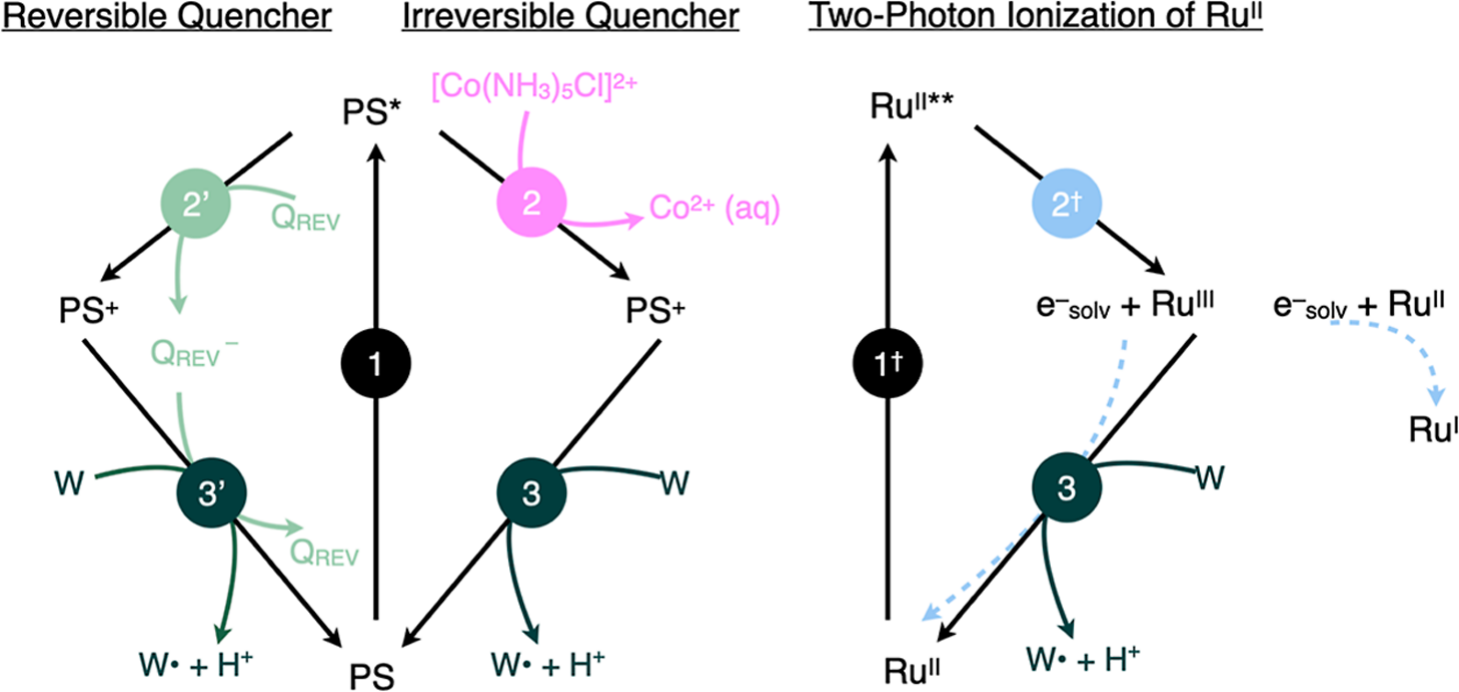
Generation of W^•^ by flash-quench photolysis using a reversible quencher (left), an irreversible quencher (middle), or two-photon ionization (right); (1) represents laser excitation of the photosensitizer (PS = [Ru(dmb]_3_]^2+^ or [ZnTPPS]^4–^), which is followed by oxidative quenching by an irreversible quencher (2), a reversible quencher (2′), or ionization (2^†^). Recovery of the photosensitizer to the ground state (3) either via reaction with a W analogue or via a combination of reaction with W and recombining with the electron lost in the oxidation process.

The photoinitiated reactions involved in W-analogue oxidation are shown in Figure 3 for three different oxidative quenching scenarios: reversible quenching, irreversible quenching, and sequential two-photon ionization without a quencher. In all three scenarios, step 1 is the excitation of the photosensitizer (PS) by a laser flash, step 2 involves oxidative quenching of the excited photosensitizer to produce PS^+^, and step 3 is the oxidation of the W analogue by PS^+^. Below, we discuss each of these approaches for resolving the rates of W-analogue oxidation.

Reversible quenchers, methyl viologen (MV^2+^) or [Ru(NH_3_)_6_]^3+^, were utilized in the acquisition of data shown in Figure 2.^7,22^ Under reversible quenching conditions, the reduced quencher (MV^•+^ or [Ru(NH_3_)_6_]^2+^) can undergo charge recombination with the oxidized photosensitizer (step 3′, Figure 3). When rates of W-analogue oxidation are slow, for example, at low pH, the charge recombination reaction will compete for oxidizing equivalents from PS^+^, effectively reducing the yield of the oxidized W analogue. As the rate of charge recombination approaches the rate of W-analogue oxidation, the observed kinetics will increasingly reflect the rates of recombination. This effect gives a greater uncertainty in the determination observed W-analogue oxidation rates. The disagreement in WEE oxidation rate constants at low pH in Figure 2 could be explained by such uncertainties.

MV^2+^ and [Ru(NH_3_)_6_]^3+^ exhibit limitations for use as quenchers in these studies. It was suggested that MV^2+^ forms an adduct with WEE at high concentrations (25 mM of each species) and high pH (∼10 and above).^7^ This suggestion was supported by the appearance of a new optical absorption at 400 nm at high concentrations of MV^2+^. The tendency to form a MV^2+^-WEE adduct was not investigated under the conditions of the kinetic experiments, specifically with lower MV^2+^ concentrations, and it was not possible to quantify what, if any, influence this had on the WEE oxidation.^7^ To avoid potential complications with an adduct at higher pH, [Ru(NH_3_)_6_]^3+^ was used as the reversible electron acceptor in ref (7). Use of [Ru(NH_3_)_6_]^3+^ as a quencher is limited by its instability at high pH.^7,29^ We could confirm that at pH > 8, aqueous solutions of [Ru(NH_3_)_6_]^3+^ rapidly turn black, showing loss of molecular integrity. The oxidative quenching reaction between *[Ru(dmb)_3_]^2+^ and [Ru(NH_3_)_6_]^3+^ at high pH is not well defined; this uncertainty could lead to errors in the determination of oxidation rate constants from kinetic fits.

In this investigation, we avoid the limitations described above by using an irreversible electron acceptor, [Co(NH_3_)_5_Cl]Cl_2_, over a pH range of 2–7.5. Use of an irreversible quencher eliminates the kinetic complications from charge recombination and is particularly useful at the lower pH values where the WEE oxidation rates are expected to be slow. Upon reduction step 2 (Figure 3), [Co(NH_3_)_5_Cl]Cl_2_ decomposes to Co^2+^ (aq), NH_4_^+^ (aq), and Cl^–^ (aq), which leads to a slight increase in pH of the solution with each laser flash (roughly 0.5 pH units per 5–10 laser flashes). Each TA experiment was composed of 4–8 laser flashes. The change in pH was monitored before the first flash and after the last flash; the reported pH is the average of these two measurements. [Co(NH_3_)_5_Cl]Cl_2_ could not be used throughout the entire pH range because it is a relatively slow quencher (pseudo-first-order rate constant of quenching *k*_q_ ≈ 3.6 × 10^6^ s^–1^ for reaction with *[Ru(dmb)_3_]^2+^), and its solubility is also pH-dependent and limited to ∼10 mM (with higher solubility at high pH).^30^ At high pH values, this results in a quenching reaction that occurs on a similar timescale to WEE oxidation, meaning that the latter will not be resolved and observed.

At pH > 7.5, the photosensitizer [Ru(dmb)_3_]^2+^ was instead oxidized in the absence of a quencher via sequential two-photon excitation at 355 nm (Figure 3).^31−34^ During the photoionization, freely solvated electrons are formed within the duration of the 10 ns laser pulse. The solvated electrons exhibit broad absorption with a peak centered around 700 nm and a lifetime of ∼0.1 ms before they recombine with [Ru(dmb)_3_]^2+^ or [Ru(dmb)_3_]^3+^.

Recovery of the [Ru(dmb)_3_]^2+^ ground state after each laser shot was complete and occurred on a time scale of ∼100 μs in all experiments at pH > 7.5 (Supporting Information page S11). The oxidized sensitizer in the absence of WEE is stable on much longer time scales than that, even in alkaline water. The stronger oxidant [Ru(bpy)_3_]^3+^ (ruthenium(II)-2,2′-bipyridine) has been reported to decay by reactions with OH^–^ with a rate constant of *k* = 148 M^–1^ s^–1^ at pH > 11.^35^ This would correspond to a Ru(III) lifetime on the order of 1 s at pH = 11.4, which is clearly much slower than what is observed here in the two-photon ionization experiments.

For this study, all TA experiments were carried out in 0.5 mM KP_i_ buffer. This concentration has been shown to be small enough such that water, and not the buffer, acts as the primary proton acceptor.^22^ Unless otherwise specified, the kinetic traces of [Ru(dmb)_3_]^2+^ (ground state) recovery were fit to a single exponential for the model of a pseudo-first-order reaction with excess WEE or NAWEE (vide infra). The second-order rate constants were determined by dividing the pseudo-first-order rate constants obtained from kinetic fits by the W-analogue concentration present in the experiment.

### 2.4 WEE Oxidation is Coupled to Proton Transfer

**Figure 4:**
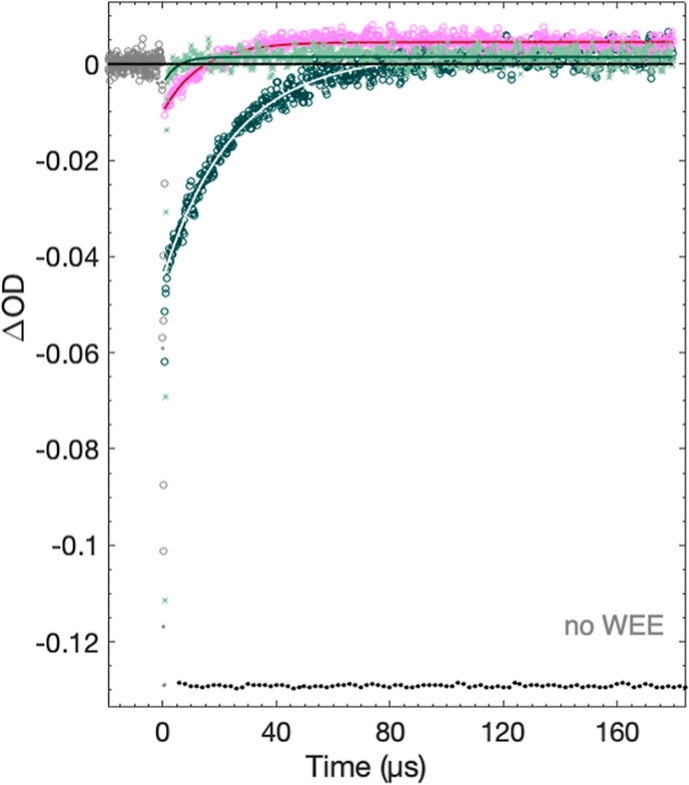
TA kinetic traces (symbols) and single-exponential fits (solid lines) after laser pulse excitation at 460 nm of a solution of 20 μM [Ru(dmb)_3_]^2+^, 5 mM WEE, and 4 mM [Co(NH_3_)_5_Cl]^2+^ obtained in pH 6.0 (±0.1) 0.5 mM KP_i_ at 510 nm (pink circles; magenta line), 560 nm (light-green crosses; dark-green line), and 450 nm (dark-green circles; white line). Black dots indicate the control experiment obtained at 450 nm without WEE and normalized to the 450 nm bleach.

The rate constants for WEE oxidation were obtained as a function of pH from pH 2.0 to 11.4 using [Ru(dmb)_3_]^2+^ as the photosensitizer. At pH < 7.5, [Ru(dmb)_3_]^2+^ was excited at 460 nm in the presence of the [Co(NH_3_)_5_Cl]Cl_2_ quencher, while at pH > 7.5, [Ru(dmb)_3_]^2+^ was excited by 355 nm laser light, resulting in two-photon ionization. Kinetic data were obtained from transient absorption measurements at different wavelengths, Figure 4. Monitoring the reaction at 450 nm allowed us to follow the bleach and recovery of the [Ru(dmb)_3_]^2+^ ground state. Recovery of the signal at 450 nm back to the baseline can be unambiguously assigned to the oxidation of WEE (step 3, Figure 3); in the absence of WEE, the signal at 450 does not recover, indicating that no competitive recombination reactions occur on experimental timescales. The formation of the neutral radical, WEE^•^, was indicated by the appearance of a positive signal at 510 nm^6,9^ with comparable kinetics to the recovery of the 450 nm signal. Protonated tryptophan radicals, W^•+^, exhibit an absorption maximum at 560 nm. At low pH (≤3), a strong signal at 560 nm indicated the formation of WEE^•+^, vide infra. At higher pH values (≥5), a very weak positive signal at 560 nm was observed in kinetic traces for WEE oxidation, Figure 4. Based on the small amplitude of the 560 nm signal, however, it can be attributed to the shoulder of the neutral radical absorption peak.^6,9^ These data show that the oxidation reaction pH ≥ 5 involves the loss of both an electron and a proton from WEE, confirming that WEE oxidation proceeds by PCET. This is expected as p*K*_a_(W^•+^) is ∼4.3 (see above).

**Figure 5:**
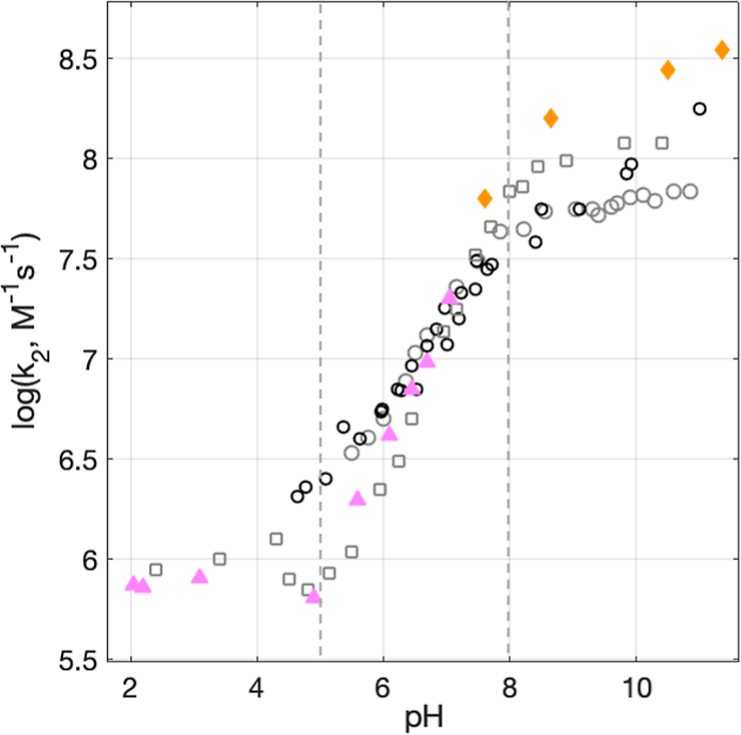
Experimental PCET rate constants for WEE oxidation by [Ru(dmb)_3_]^3+^ as a function of pH. Filled pink triangles represent data collected using [Co(NH_3_)_5_Cl]^2+^ as a quencher. Filled orange diamonds represent data with two-photon ionization without acceptors. Data in gray from ref (7), black data from ref (22), are reproduced from Figure 2. Circles represent data collected using MV^2+^ as quencher. Squares represent data collected using [Ru(NH_3_)_6_]^3+^ as a quencher. The dashed vertical lines mark pH regions defined in the text.

The PCET rate constants determined for WEE as a function of pH in the present study are shown in Figure 5 as filled triangles and diamonds. Previously reported data are shown as open squares and circles.^7,18^ The filled data are discussed in detail below. Single-shot kinetic traces with fits and residuals for each data point are found in the Supporting Information.

#### 2.4.1 pH-dependent Rate Constants for WEE Cannot be Explained by an ET-Limited Reaction

New data from the present study are discussed along with the previously published results^7,22^ in the following pH regions: pH < 4, pH 5–8, and pH > 8.

At pH < 4. pH below the p*K*_a_ of oxidized WEE (p*K*_a_ = ∼4.3 for W^•+^/W^•^), no deprotonation of the oxidized WEE radical is expected. From the data in Table 1, one may assume that Δ*G*°_ET_ > 0, but WEE is in great excess of [Ru(dmb)_3_]^3+^, which can drive the reaction. Kinetic traces in this pH region show a larger positive signal at 560 versus 510 nm, indicating the formation the protonated radical, WEE^•+^, Figure S7. The kinetic traces at 450 nm probed the recovery of the [Ru(dmb)_3_]^2+^ ground-state bleach. With a high concentration of WEE (80 mM), these were single-exponential, indicating a pseudo-first-order reaction, with a rate constant that agreed well with previously published results^7^ (Figure 5). At lower concentrations of WEE, the kinetics were not single-exponential; instead, the data are consistent with an uphill pre-equilibrium ET (Δ*G*°_ET_ > 0), followed by further reactions of the radical cation (e.g., dimerization); see Supporting Information, page S5, for further details. Thus, while the observed rate constant is the sum of those for ET and its reverse, the concentration of WEE at 80 mM is sufficiently large that the forward rate constant dominates and that this gives a sufficiently precise determination of *k*_ET_.

5 < pH < 8. In this region, all previously published data exhibit rate constants that change as a function of pH with a slope < 1, seen in Figure 5. Between pH ∼ 5 and 6, previous data do not agree well depending on which quencher, MV^2+^ or [Ru(NH_3_)_6_]^3+^, was used, where PCET rate constants obtained with MV^2+^ as the quencher exhibited a weaker slope. The present data at pH 5–8 agree best with PCET rate constants determined with [Ru(NH_3_)_6_]^3+^ as the quencher and have a slope of ∼0.7 (a fit is shown in Figure 8, vide infra).

pH > 8. In this pH region, the previously published WEE oxidation rate constants are not in agreement. Specifically, data from ref (22) with MV^2+^ as the quencher appear to increase with increasing pH, while data from ref (7) appear to level out after pH ∼ 8 with both MV^2+^ and [Ru(NH_3_)_6_]^3+^ as quenchers, but at different rate constant values. For the present data, we wanted to avoid using MV^2+^ and [Ru(NH_3_)_6_]^3+^ quenchers because of their respective reported issues at high pH values (vide supra). [Co(NH_3_)_5_Cl]^2+^ could not be used due to its relatively slow quenching rate constant. The [Ru(dmb)_3_]^3+^ species was therefore instead generated via direct two-photon-induced electron transfer to water (photoionization) without the use of any quenchers. The photoionization occurs when [Ru(dmb)_3_]^2+^ sequentially absorbs two photons at 355 nm during the same ∼10 ns laser pulse (step 1 and 1^†^ in Figure 3, right side).^31−34^ The solvated electrons formed have a broad absorption signal that overlaps with the WEE^•^ at 510 nm. At 510 nm, a large positive signal is seen, followed by a decay as the electrons recombine with either [Ru(dmb)_3_]^2+^ or [Ru(dmb)_3_]^3+^. A WEE concentration of 0.1 mM was used in the pH region studied. At concentrations greater than 0.1 mM, mixed kinetics between [Ru(dmb)_3_]^3+^ formation and WEE oxidation were observed. At 0.1 mM WEE, there was a small component of competitive recombination between e^–^_solv_ and [Ru(dmb)_3_]^3+^. The kinetic contribution of WEE oxidation was extracted by the following procedure: the signal observed at 510 nm where the solvated electrons absorb was fitted to a single exponential and was then subtracted from the single-exponential fit at 450 nm which follows the recovery of the [Ru(dmb)_3_]^2+^ species. Further details can be found in Supporting Information page S11. The rate constants of WEE oxidation are reported in Figure 5 as orange filled diamonds. The rate constants of WEE oxidation by the two-photon ionization method do not level off with increasing pH and agree well with those determined in ref (22). This shows that the pH dependence of the rate constant for WEE oxidation cannot be explained by a simple titration of the amine group since the rate constants continue to increase at pH values well beyond the amine p*K*_a_ at ∼7.5. It also shows that WEE oxidation cannot occur by ETPT in the entire pH range as the observed rate constant for stepwise ETPT cannot exceed that for the initial ET step, which was measured at pH < 4 (*k* = 1 × 10^6^ M^–1^ s^–1^).

To further exclude any effects of side group titration, we examined the oxidation of NAWEE, where both side groups are protected.

### 2.5 NAWEE Exhibits pH-dependent Rate Constants with a Weaker Oxidant

When NAWEE oxidation was studied with [Ru(dmb)_3_]^3+^ as the photogenerated oxidant under reversible quenching conditions, pH-independent oxidation rate constants were observed.^7^ For this study, we redetermined the rate constants of NAWEE oxidation using the same oxidant, [Ru(dmb)_3_]^3+^, and [Co(NH_3_)_5_Cl]^2+^ as a sacrificial quencher and could confirm this result, Figure S16, Supporting Information. The pH independence of oxidation rate constants is consistent with the lower reduction potential for NAWEE in comparison to WEE, Table 1. The lower reduction potential for NAWEE favors an electron-transfer-limited ETPT mechanism when [Ru(dmb)_3_]^3+^ is used (Point 3, Introduction). The use of a weaker oxidant would open the possibility for NAWEE oxidation that proceeds by a CEPT mechanism.^12^ To test this hypothesis, we used a weaker oxidant, [ZnTPPS]^3–^, where *E*°([ZnTPPS]^4–/3–^) = +0.87 V versus NHE (Table 1), as a photogenerated oxidant.

**Figure 6:**
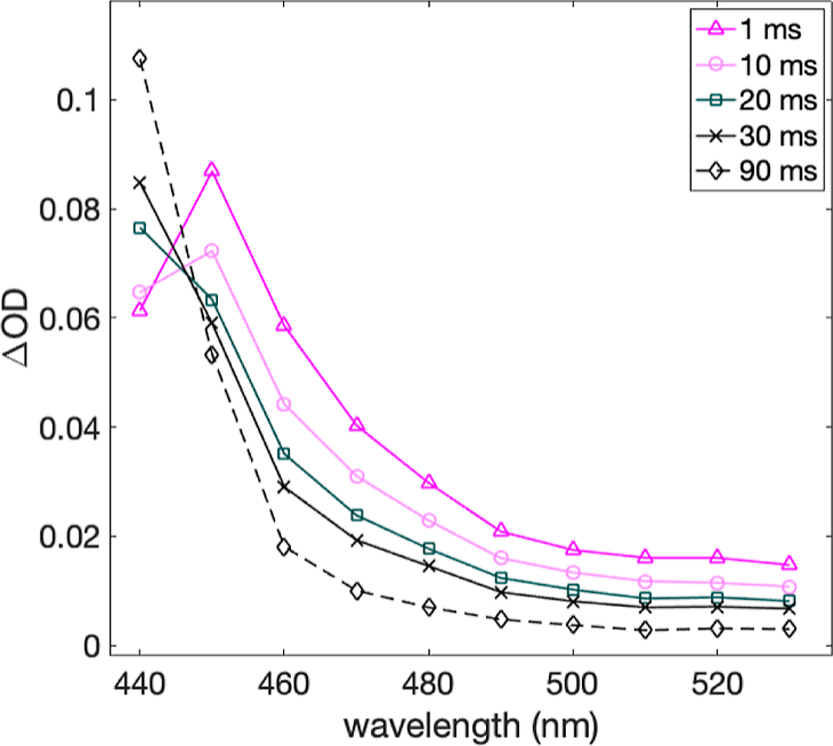
Transient spectra of 20 μM [ZnTPPS]^4–^ after laser pulse excitation at 545 nm in the presence of 0.53 mM [Co(NH_3_)Cl]^2+^ and 0.42 mM NAWEE in 0.5 mM KP_i_ buffer at pH = 7.8. Spectra were constructed from single-wavelength traces with a fresh sample for each laser shot.

**Figure 7:**
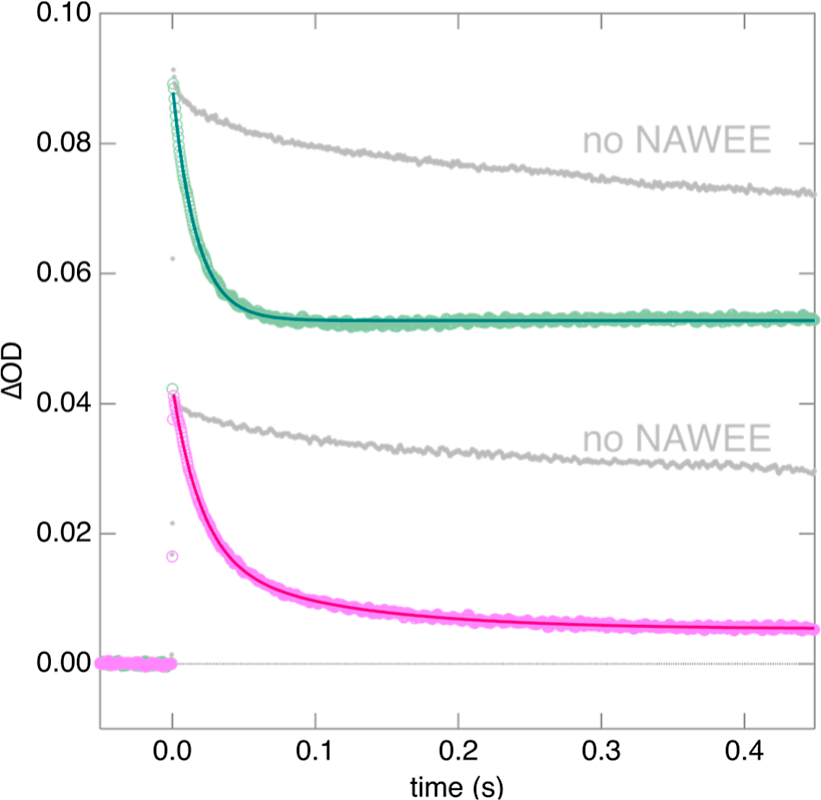
TA kinetic traces after laser pulse excitation at 545 nm 20 μM [ZnTPPS]^4–^, 0.42 mM NAWEE, and 0.53 mM [Co(NH_3_)_5_Cl]^2+^ collected at pH = 7.8 ± 0.2 in 0.5 mM KP_i_ at 450 nm (upper trace, green data with a dark single-exponential fit) and 470 nm (lower trace, pink data with a magenta double-exponential fit). Gray dots are control experiments collected at 450 and 470 nm without NAWEE.

Using the same laser flash-quench method as above, [ZnTPPS]^4–^ was excited in the Q-band at 545 nm^35^ and its triplet state was oxidatively quenched by [Co(NH_3_)_5_Cl]^2+^ on the timescale of ∼200 ns (Figure S18). The transient absorption spectrum shows a peak at 450 nm from the resulting [ZnTPPS]^3–^ radical.^36^ In absence of NAWEE, the radical is stable up to at least several seconds (Figure S18). In the presence of NAWEE, this absorption peak disappears on the ms timescale; Figure 6 shows data at pH = 7.8, where the [ZnTPPS]^3–^ absorption seen after 1 ms is gone after 30 ms. The corresponding kinetic trace at 450 nm (Figure 7) shows a single-exponential decay with a time constant of 17 ms at 0.42 mM NAWEE (pseudo-first-order conditions). A peak at ca. 510 nm from the resulting NAWEE^•^ radical was not observed. This is not unexpected since tryptophan radicals are known to undergo rapid dimerization in solution.^10^ A sufficiently rapid rate of dimerization would lead to amounts of NAWEE^•^ so small that they are undetectable. Instead, we see a remaining, indistinct positive absorption that decreases slightly at >450 nm on a 100 ms time scale but is thereafter quite stable. Indeed, a steady-state absorption spectrum of the solution taken after laser exposure shows significant buildup of a photoproduct of the oxidized porphyrin already after a single flash (Figure S17). For this reason, all data used for kinetic analysis were obtained with fresh samples for each laser flash, carefully avoiding light exposure prior to the experiment. The slower spectral evolution conveniently showed an isosbestic point at the [ZnTPPS]^3–^ maximum at 450 nm, resulting in single-exponential traces representing the PCET reaction. The kinetic traces at 470 nm instead were fitted with a biexponential function where the fast component had the same lifetime as that at 450 nm, and the slow component, with a comparatively small amplitude, had a lifetime of 103 ms at pH = 7.8. This shows that the PCET reaction and the much slower porphyrin degradation are kinetically well separated, and the latter should not interfere with analyses of the former.

**Figure 8:**
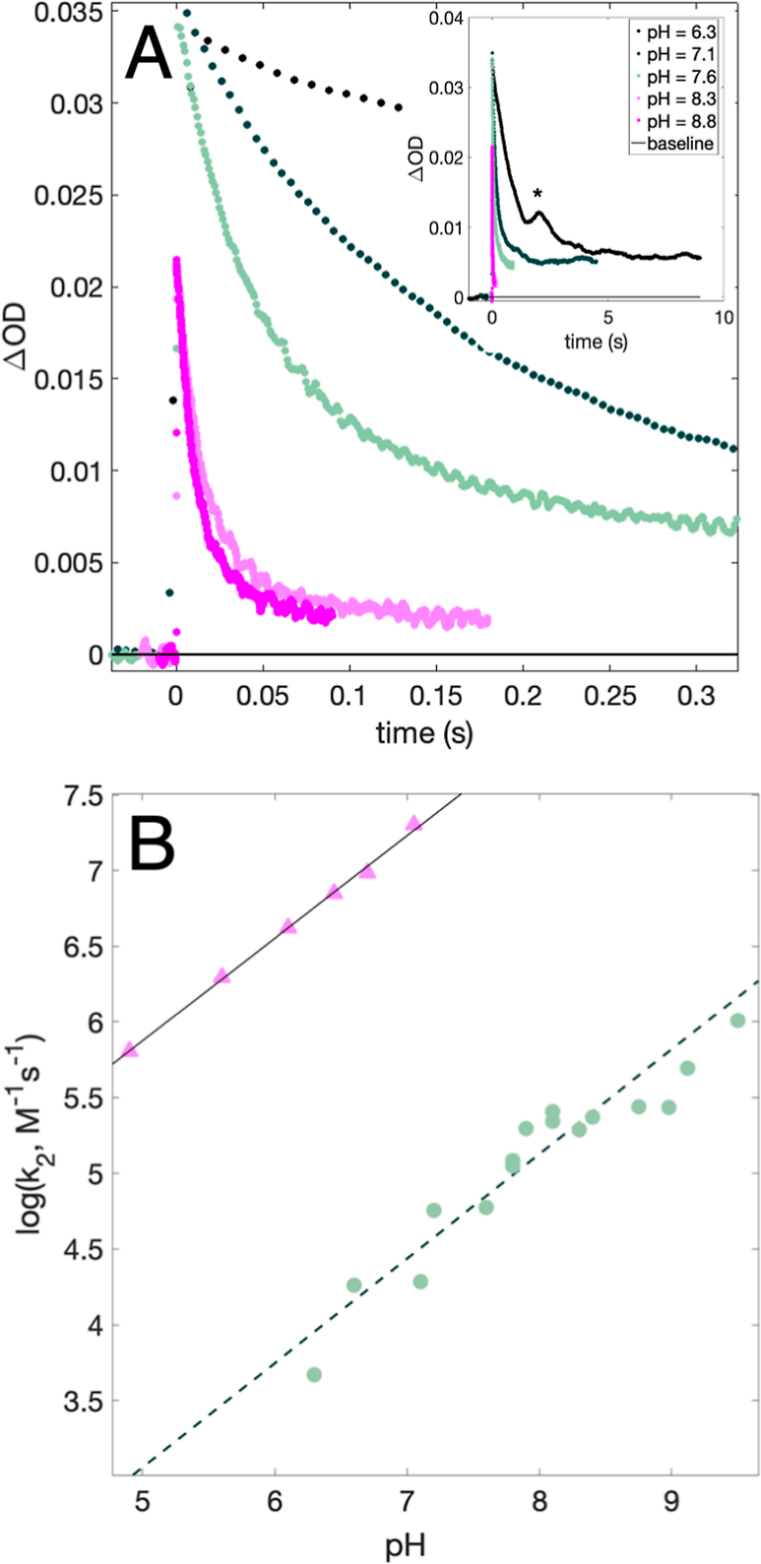
(A) TA kinetic traces recorded at pH = 6.3–8.8 on samples containing 0.39–0.42 mM NAWEE, 20–21 μM [ZnTPPS]^4–^, and 0.4–0.5 mM [Co(NH_3_)_5_Cl]^2+^. The inset shows the same traces on a longer timescale; the asterisk (*) denotes a probe lamp artifact. (B) Experimental second-order PCET rate constants for NAWEE oxidation by [ZnTPPS]^3–^ (green dots). The dashed black line represents a linear fit corresponding to *f*(*x*) = 0.69*x*–0.39. Also shown are PCET rate constants for WEE obtained using [Ru(dmb)_3_]^2+^ as the photosensitizer and [Co(NH_3_)_5_Cl]^2+^ as the quencher (pink triangles), reproduced from Figure 5. A fit to *f*(*x*) = 0.68*x* + 2.5 is shown as a solid black line.

The rate constants for NAWEE oxidation were thus obtained from biexponential fits to traces collected at 470 nm, Figure 8A. Figure 8B summarizes these second-order rate constants (*k*_2_) as a function of pH (green dots). For comparison, the rate constants for WEE oxidation by [Ru(dmb)_3_]^3+^ as a function of pH are shown (pink triangles). The oxidation of both NAWEE and WEE shows a linear dependence of log *k*_2_ on pH with a slope of 0.7. This shows that a similar pH dependence to that observed for WEE can be obtained even in the absence of a protonatable amine group. The mechanistic implications of these results are discussed in the next section.

### 2.6 General Discussion

#### 2.6.1 CEPT as a Mechanism for WEE and NAWEE Oxidation

The pH dependence observed for NAWEE and [ZnTPPS]^3–^ is the same as for the reactions of [Ru(dmb)_3_]^3+^ and WEE, which we have been assigned to a CEPT reaction.^22^ Our results exclude the stepwise reactions. PTET would have given a slope = 1 in Figure 8B, and because of the large p*K*_a_ value of W (∼17), the fraction of W^–^ would be too small to produce the observed rate constants. For example, at pH = 7, the fraction is only [W^–^]/[W] ≈ 10^–10^. For a pre-equilibrium PTET reaction via the W^–^ fraction, only 10^–10^ of the encounters will lead to reaction, which with a diffusion-controlled encounter (∼10^10^ M^–1^ s^–1^) would give a rate constant as small as *k*_PTET_ ∼ 1 M^–1^ s^–1^. This is 7 and 4 orders of magnitude lower than the observed values for WEE and NAWEE, respectively. PT-limited PTET in the encounter complex would also be too slow to account for the observed rate constants (see the Supporting Information, page S28). An ETPT mechanism, on the other hand, can be excluded because the observed rate constant cannot be larger than that for the forward ET step which for WEE was measured at low pH (1 × 10^6^ M^–1^ s^–1^). Also for NAWEE with [Zn(TPPS)]^3–^, an ETPT would have been slower than what is observed; the oxidant is much weaker than [Ru(dmb)_3_]^3+^, but the observed rate constant at high pH nevertheless reaches a value similar to that for ET in WEE. This would leave CEPT as the plausible mechanism for the observed reactions at pH > 5 for both WEE with [Ru(dmb)_3_]^3+^ and NAWEE with [ZnTPPS]^3–^. The very similar pH dependence of the two systems suggests that they follow the same mechanism. At pH < 4, the observed reaction for WEE is single ET with a comparatively slow rate (Figure 5).

#### 2.6.2 Water as the Primary Proton Acceptor for CEPT

The kinetic data indicate that WEE and NAWEE oxidation proceeds by CEPT. The TA data show that the electron is transferred from WEE or NAWEE to the oxidized photosensitizer. For WEE, the formation of WEE^•^ was directly observed to be concomitant with the recovery of the [Ru(dmb)_3_]^2+^ signal (cf. Figure 4). The initial proton-transfer step involves a buffer, water, or OH^–^. The rate constants for PCET in WEE and NAWEE were determined in buffers of a sufficiently low phosphate concentration (0.5 mM) such that it did not act as the primary proton acceptor. This can be clearly seen from control experiments with varying concentrations of the buffer, which for WEE and [Ru(dmb)_3_]^3+^ were published in ref (22). Table S8 gives rate constants for NAWEE oxidation by [ZnTPPS]^3–^ at pH ∼ 7.9 for buffer concentrations ranging from 0.5 to 5 mM KP_i_. No significant trend in rate constant with buffer concentration was observed, and it can be concluded that at 0.5 mM, KP_i_ plays a minimal role as the primary proton acceptor. This leaves water or OH^–^ as the possible primary proton acceptor under the conditions examined.

To determine to what extent OH^–^ may act as the proton acceptor in a CEPT reaction, we estimated the upper limit for that rate constant by assuming that CEPT in the oxidant/W-analogue encounter complex is controlled by its diffusional encounter with OH^–^ (*k*_diff_ ∼ 10^10^ M^–1^ s^–1^ in water). The formation of the encounter complex was assumed to be thermoneutral (association constant *K* ∼ 1; see Supporting Information page S27 for the full derivation). From this estimate, we found that with WEE at pH < 12, the [OH^–^] is too small to agree with the observed rate constants. For NAWEE, with slower oxidation rates, [OH^–^] is too small at pH < 10. With these estimates, PCET with OH^–^ as a primary proton acceptor cannot explain the pH-dependent rate constants observed. Furthermore, if OH^–^ acted as the primary proton acceptor, the observed rate constant should increase by a factor of 10 per pH unit, instead of the weaker pH dependence observed. Thus, water remains as the only viable primary proton acceptor in the pH range studied.

#### 2.6.3 pH-Dependent CEPT Rate Constants for Radical Formation

The weak pH dependence of the PCET rate constants can be incorrectly believed to reflect a Marcus-type free-energy dependence of the rate constant on the free energy of the overall process.^37^ However, as mentioned in the Introduction section, CEPT rate constants should not be pH-dependent when H_2_O is the primary proton acceptor. The apparent pH dependence of the potential of a proton-coupled redox process (*E*°′), as reflected in Pourbaix diagrams, is due to the increase in mixing entropy of the proton with pH. The driving force for the elementary PCET process is, however, independent of pH when water is the primary proton acceptor. Similar to the case for protonation of photoacids, a small cluster of water molecules acts as the primary acceptor, and the conjugate acid, that is, the solvated proton (H_3_O^+^_aq_), has p*K*_a_ ≡ 0. The dilution of the proton in the bulk water becomes more exergonic with increasing pH, but this process is not expected to influence the measured rate of the PCET reaction. Thus, the elementary CEPT step should not be pH-dependent, as was pointed out by Sjödin et al. already in 2005^9^ before publication of refs (20) and (21) and clearly stated in all published papers on the topic from our group since then. In contrast to what is stated in ref (7), we agree on this point. However, our group has published several studies with experimental data showing a similarly weak pH dependence (slope < 1 of a log *k* vs pH plot) of PCET rate constants for several tryptophan and tyrosine derivatives,^6,22,23,37−39^ a dependence that currently lacks a theoretical explanation. This present study adds to that list. The [Ru(dmb)_3_]^3+^/WEE system exhibits pH-dependent rate constants that exceed those limited by ET measured at low pH. Similarly, we can exclude a PTET mechanism because of the large p*K*_a_ value of WEE and NAWEE. This strongly suggests a CEPT reaction with water as the primary proton acceptor.

#### 2.6.4 How Water Acts as a Proton Acceptor in PT and PCET

The dynamics of acid deprotonation to H_2_O has been studied extensively, particularly using photoacids. In the widely accepted model of Eigen and Weller,^40,41^ the excited acid, HA, with p*K*_a_ > 0 undergoes an initial proton-transfer step to form an ion pair, stabilized by solvent fluctuations (eq 3). This reversible, endergonic step is followed by dissociation and solvent cage escape to form the free base, A^–^, and hydronium, H_3_O_aq_^+^
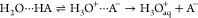
3

The fractional population of the intermediate [H_3_O^+^···A^–^] state depends on the p*K*_a_ value of the acid where an increase in one p*K*_a_ unit leads to a 10-fold decrease in fractional population. It follows that the deprotonation rate constant decreases 10-fold per one p*K*_a_ unit. This trend in deprotonation rate constants as a function of p*K*_a_ has been experimentally observed.^40−42^ pH-dependent rate constants for an individual acid are in general not observed so long as H_2_O is the primary proton acceptor.

For the present CEPT reaction of tryptophan and its analogues (W–H), one may set up a similar model, where the initial reversible step is coupled to an electron transfer, eq 4.^6^ Here, the intermediate product is not an ion pair but has the charge-neutral W^•^; this does not change the basic picture outlined in eq 3. The external electron acceptor, for example, [Ru(dmb)_3_]^2+^, takes the electron from [H_2_O···W–H] in concert with proton transfer
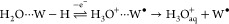
4

The first step is endergonic because of the high p*K*_a_ of the radical cation and a slightly lower *E*° for Ru^III/II^ than for W^•+/0^. Just as for acid deprotonation, the CEPT reaction is driven by the dissociation and cage escape of the proton.

For an ETPT mechanism, the initial ET step is endergonic, and a situation analogous to CEPT should result
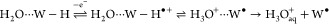
5

Pre-equilibrium ET and PT lead to the same intermediate [H_3_O^+^···W^•^] state as in CEPT, which is followed by proton dissociation and cage escape. With the CEPT and ETPT models, the fractional population of the [H_3_O^+^···W^•^] intermediate is the same, irrespective of the mechanism, which means that CEPT would not be disfavored relative to ETPT by the high p*K*_a_ of W^•^H^+^. This picture is different from previous predictions that are discussed in the Introduction section.^20,21^ Since the CEPT reaction requires both sufficient electronic coupling and wave function overlap between reactant and product states, CEPT could come at a kinetic disadvantage compared to stepwise mechanisms. On the other hand, the reorganization energy may be smaller for CEPT as no charge is formed on the W unit. Importantly, our present and previous experiments show that CEPT can indeed be competitive for W in water.^6,9,22,23^

The above description of water acting as the primary proton acceptor in PCET reactions is not able to explain the observed pH-dependent rate constants nor the fact that the mechanism changes from ETPT at low pH to CEPT at neutral and higher pH. This would suggest that our present knowledge and models for something as fundamentally important as deprotonation in water are incomplete as it is yet not possible to fully capture the behavior of PCET reactions.

## 3 Conclusions

There is consensus that among Nature’s 20 amino acids, the most recently evolved members are cysteine, methionine, tyrosine (Y), tryptophan, and selenocysteine. The appearance of these amino acids coincides with the increase of oxygen in the Earth’s atmosphere. The above amino acids are more redox-accessible, making them better suited to protect enzymes from oxidative damage.^43^ Chains of Y and W residues have been identified in approximately one-third of the protein structures available in the Protein Data Bank, which points to their importance in natural systems where they can facilitate long-range electron and radical transfer.^3^ How W and Y function in Nature is still not fully understood, although studies of natural and model systems have provided much insight. Compared to W, Y has a p*K*_a_ value of 10 in its reduced form and −2 in its oxidized form;^5^ this allows more facile deprotonation of Y. In a protein environment, Y exhibits a higher reduction potential, *E*°(Y^•+^/Y) = 1.510 V, compared to *E*°(W^•+^/W) = 1.293 V.^10^ This permits easier oxidation for W compared to Y. We have recently shown that Y buried in a hydrophobic protein environment can undergo PTET and CEPT with water as the most likely primary proton acceptor.^44^ Similar behavior has been suggested for Y at the interface between two subunits in Class 1 RNR.^45,46^ CEPT from W with water as the primary proton acceptor, discussed in the Introduction section, is less viable due to the much larger p*K*_a_ values of the reduced and oxidized forms. The large family of DNA photolyase and cryptochrome enzymes has a common three-W motif where one W is in a solvent-exposed position and has been shown to undergo ETPT.^47−49^ In contrast, the present paper shows that W can undergo CEPT with water as the primary proton acceptor. While CEPT in W has yet to be directly observed in natural systems, our results show that PCET in a surface-exposed W is not limited to ETPT or pure ET by default.

Our investigation of WEE and NAWEE has brought further clarity to the PCET mechanisms in tryptophan model systems. First, we have shown that the electrostatic effects of side-group deprotonation in WEE are negligibly small and do not provide a significant contribution to the pH-dependent rate constants. Second, the change in rate constants as a function of pH indicates that there must be a change in mechanism from an ET-limited mechanism at low pH to another mechanism at higher pH. The mechanism at higher pH values (at pH > p*K*_a_ of oxidized WEE) is likely CEPT. In some proteins, hydrogen bonding interactions have been shown to affect the reduction potential for tyrosine and tryptophan.^50,51^ This could result in changes in apparent reduction potential with pH that are different from the expected 59 mV/pH unit of a Pourbaix diagram for a 1 electron/1 proton couple. However, such a trend has not been observed in experimental Pourbaix diagrams of freely solvated tryptophan or tryptophan in small synthetic proteins, which do not deviate from the 59 mV/pH unit slope.^10,25^ Therefore, this cannot explain the observed pH dependence. Third, we have determined the reduction potentials of WEE and NAWEE and found that the latter is easier to oxidize. This explains the lack of pH dependence reported by ref (7) when studying NAWEE with [Ru(dmb)_3_]^3+^ as the oxidant. With this oxidant, the reaction can be assigned to an ET-limited ETPT mechanism which should not exhibit pH-dependent rate constants. Fourth, we have also shown that when we switch to a weaker oxidant, [ZnTPPS]^3–^, NAWEE exhibits pH-dependent rate constants that parallel those of WEE with [Ru(dmb)_3_]^3+^. This is the same behavior observed for Ru-W dyads that switch mechanism according to the oxidant strength.^6,9,23^ Importantly, this shows that the observed pH dependence is independent of the identity of the tryptophan analogue or the presence of protonable side groups but rather depends on the oxidant strength. This supports that both the tryptophan analogues follow a CEPT mechanism at pH > 5, with a suitably mild oxidant. Finally, our results indicate that water is the primary proton acceptor in this reaction. This is in contrast to earlier theoretical predictions that tryptophan with its relatively high radical p*K*_a_ value (∼4.3) would not undergo CEPT with water as the primary acceptor but rather be restricted to ETPT or ET to generate the radical cation. This means that tryptophan CEPT with water as the primary proton acceptor is viable and should be considered when studying surface-exposed W in both synthetic and natural systems.

## 4 Materials and Methods

### 4.1 Electrochemistry

Measurements were made in 0.1 M KNO_3_ and 0.5 mM KP_i_ at pH 5.2 using a 2 mm glassy carbon disc (CH Instruments, Inc.) as the working electrode, Ag/AgCl (4 M) suspended in a salt bridge as the reference electrode, and a Pt rod as the counter electrode. The working electrode was polished between each scan using 0.05 μm alumina paste (Buehler Micropolish II) and then rinsed with water. The cell resistance (Ohmic drop) was determined using the NOVA program to 120 Ω, and a 90% compensation was used (108 Ω). Cyclic voltammograms were recorded with 0.2 mM tryptophan analogue at scan rates varying from 0.1 to 5 V/s using the NOVA program and Autolab PGSTAT302. Only scan rates between 0.1 and 1 V/s were used in determining the apparent redox potentials; see Supporting Information page S2 for motivation.

### 4.2 Transient Absorption Spectroscopy

Transient absorption (TA) was measured using a ns-laser pump probe setup as previously described.^44^ The sample was excited using a Nd/YAG laser (Quantel, Brilliant) passed through an OPO tuned to 460 nm for experiments with [Ru(dmb)_3_]^2+^ and 545 nm for experiments with [ZnTPPS]^4–^. The excitation energies varied from 10 to 12 mJ/pulse for [Ru(dmb)_3_]^2+^ excitation and 22–28 mJ/pulse for [ZnTPPS]^4–^ excitation. For two-photon ionization of [Ru(dmb)_3_]^2+^, no OPO was used; instead, the sample was excited by the 355 nm laser light formed after frequency tripling with an energy of about 100 mJ/pulse. The sample was probed using an unpulsed Xe arc lamp perpendicular to the excitation light. The probe light was passed through two monochromators (Applied Photophysics, pbp Spectra Kinetic Monochromator 05-109) with one before and one after the sample set to 4- and 2-mm slit openings, respectively. The signal was detected using a photomultiplier tube (PMT, Hamamatsu R928). The signal was digitized using an oscilloscope (Agilent Technologies Infiniium 600 MHz) and processed using the Applied Photophysics LKS software. TA was measured in 4 mm × 10 mm cuvettes with the probe lamp passed through the 10 mm path for WEE experiments and 10 mm × 10 mm cuvettes for NAWEE experiments.

Samples used for TA spectroscopy were dissolved in 0.5 mM KP_i_ (KH_2_PO_4_ from Sigma Life Science ≥ 99.0% purity, K_2_HPO_4_ from Acros Organics 99+% purity). pH was adjusted with 0.1 M NaOH or HCl when necessary and measured using a Metrohm 654 pH meter and a calibrated Metrohm LL Biotrode pH electrode. pH was measured before and after TA measurements, and an average was calculated to account for the change in pH when quenching by [Co(NH_3_)_5_Cl]Cl_2_. Concentrations were determined using a UV/vis spectrometer (Cary 50), with ε_555_([ZnTPPS]^4–^) = 22100 M^–1^ cm^–1^,^35^ ε_455_([Ru(dmb)_3_]^2+^) = 14 300 M^–1^ cm^–1^,^26^ and ε_280_(W-analogue) = 5500 M^–1^ cm^–1^.^52^ The concentrations used in the WEE experiments were [WEE] = 0.1–80 mM, [[Ru(dmb)_3_]^2+^] = 25–50 μM, and [[Co(NH_3_)_5_Cl]Cl_2_] = 2.5–5 mM. In the NAWEE experiments, the following concentrations were used: [NAWEE] = 0.3–0.4 mM, [[ZnTPPS]^4–^] = 20–25 μM, and [[Co(NH_3_)_5_Cl]Cl_2_] = 0.4–2 mM. In all experiments, the photosensitizer together with the tryptophan analogue was prepared separately from the quencher and the two solutions were mixed under dark conditions.

## Supporting Information Available

Cyclic voltammograms, additional absorption traces, tabulated rate constants, and all kinetic traces used to extract rate constants and tabulated rate constants (PDF).
