# Supplementary material for: Concerted and Stepwise Proton-Coupled Electron Transfer for Tryptophan-Derivative Oxidation with Water as the Primary Proton Acceptor: Clarifying a Controversy
Source: J Am Chem Soc. 2022 Apr 13;144(16):7308–19. doi: 10.1021/jacs.2c00371 (PMC9052761; doi:10.1021/jacs.2c00371)
Supplement: Supplementary file 1 — ja2c00371_si_001.pdf [file ja2c00371_si_001.pdf]

Supplementary Information to:

Concerted and Stepwise Proton-Coupled Electron Transfer for Tryptophan-derivative Oxidation with Water as the Primary Proton Acceptor: Clarifying a Controversy

Astrid Nilsen-Moe, Andrea Rosichini, Starla D. Glover, Leif Hammarström*

Department of Chemistry, Ångström Laboratory, Uppsala University, Uppsala 75120 Uppsala, Sweden.

INDEX

I. ELECTROCHEMICAL MEASUREMENTS 2
II. FLASH-PHOTOLYSIS TRANSIENT ABSORPTION OF WEE 4
i. Probing the sensitivity of PCET rate constants to electrostatic changes in solution. 5
ii. TA Kinetic Traces and Rate Constants at Low pH (< 4) 6
iii. TA Kinetic Traces and Rate Constants at Intermediate pH (5 < pH < 7) 9
iv. TA Kinetic Traces and Rate Constants at High pH (> 7) 11
III. FLASH-PHOTOLYSIS TRANSIENT ABSORPTION OF NAWEE 19
i. NAWEE oxidation with $[Ru(dmb)_3]^{2+}$ as photosensitizer 19
ii. Identifying Rate Constants Associated with NAWEE Oxidation by $[ZnTPPS]^{4-}$ 20
iii. TA Kinetic Traces Used to Determine the pH Dependence of NAWEE Oxidation 23
iv. Rate Constants for NAWEE Oxidation as a Function of Buffer Concentration 25
IV. EXCLUDING OH^- AS PRIMARY PROTON ACCEPTOR 27
i. Concerted PCET 27
ii. Stepwise PTET 28
REFERENCES 28

I. Electrochemical Measurements

Tryptophan (W) radical formation is followed by rapid radical-radical dimerization, leading to self-inhibition which results in irreversible cyclic voltammograms (CVs).[1] Self-inhibition can be reduced by lowering the substrate concentration and increasing the scan rate. If self-inhibition is sufficiently minimized, reduction potentials can be determined from the variation of peak potentials with scan rates:[2]

$$E_{\mathrm{p}} = E° + 0.903\frac{\mathrm{RT}}{\mathrm{F}} - (\mathrm{RT}\ln\frac{10}{3\mathrm{F}}) \times \log(\frac{4\mathrm{RT}k_{\mathrm{dim}}\mathrm{C°}}{3\mathrm{F}\nu}) \qquad \text{(S1)}$$

where E_{p} is the peak potential, $E°$ is the formal reduction potential, R, T, and F have the usual meaning, k_{dim} is the rate constant of dimerization, C° is the concentration of substrate, and ν is the scan rate. Eq. S1 predicts a slope of 19.7 mV in a plot of E_{p} vs. log(ν). The scan rate was varied between 0.1 and 5 V/s for a sample containing 0.2 mM the tryptophan analog WEE or NAWEE in 0.5 mM KP_i and 0.1 M KNO_3, Figure S1. This was the same range of scan rates used for a similar system.[2] At scan rates > 1 V/s the CVs started to lose their peaked shape, which was attributed to more capacitive current. We therefore decided to use scan rates from 0.1 V/s to 1 V/s. A linear fit to the apparent peak potentials in this range of scan rates yielded a slope 18 mV for both WEE and NAWEE, respectively, suggesting that self-inhibition was not significantly perturbing the CVs, Figure S2.[2]

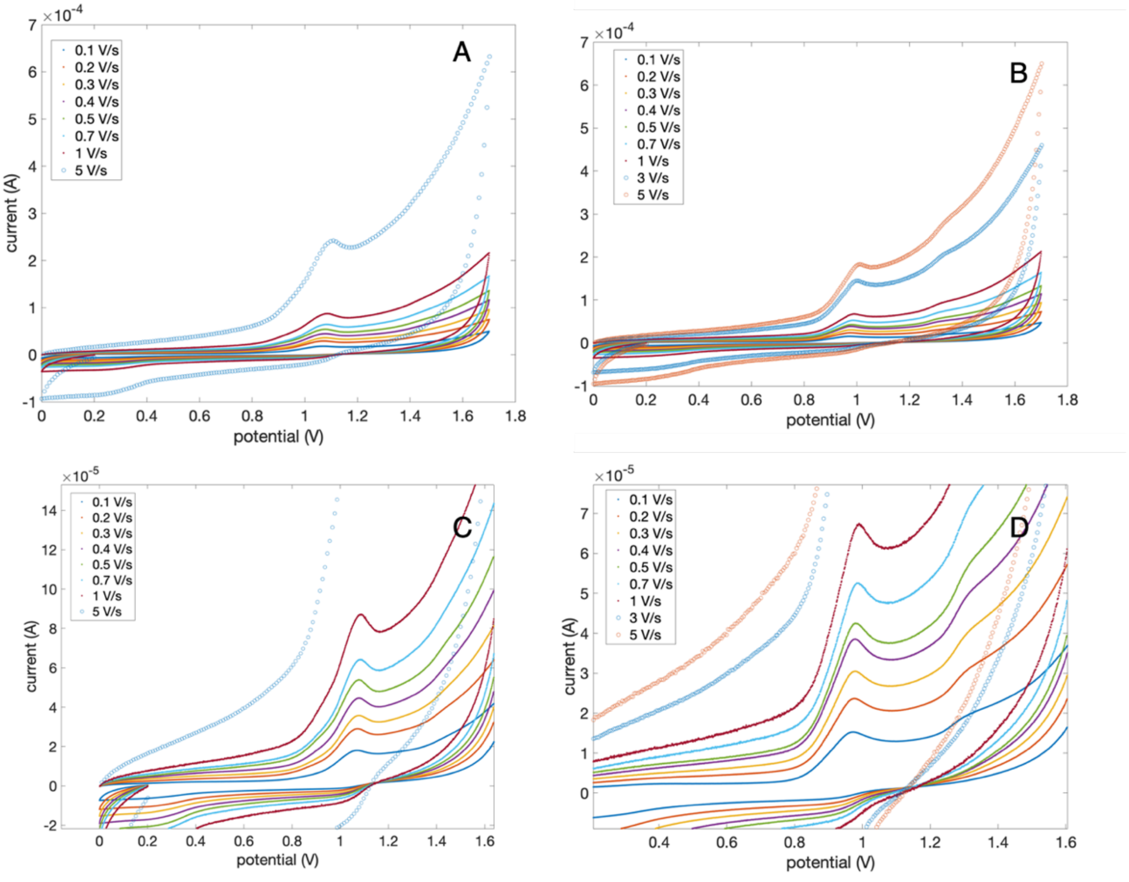

Figure S1. Cyclic voltammograms collected in 0.1 M KNO_3 and 0.5 mM KP_i at pH 5.2 using a 2 mm glassy carbon working electrode, Ag/AgCl reference electrode, and a Pt counter electrode.

Potentials are shown vs. Ag/AgCl. All peak potentials were measured vs. a Ag/AgCl reference electrode that was calibrated by measurement of the potential for the reversible $[Fe(CN)_6]^{3-/4-}$ couple (0.410 V vs. NHE).[3] A and C represent the same data collected with WEE with various zoom ins, B and D represent the same data collected with NAWEE but with different zoom ins. The black squares mark the peak potentials used in Figure S2. Conditions: 0.2 mM WEE (left panel) or NAWEE (right panel) in 0.5 mM KP_i-buffer at pH = 5.2.

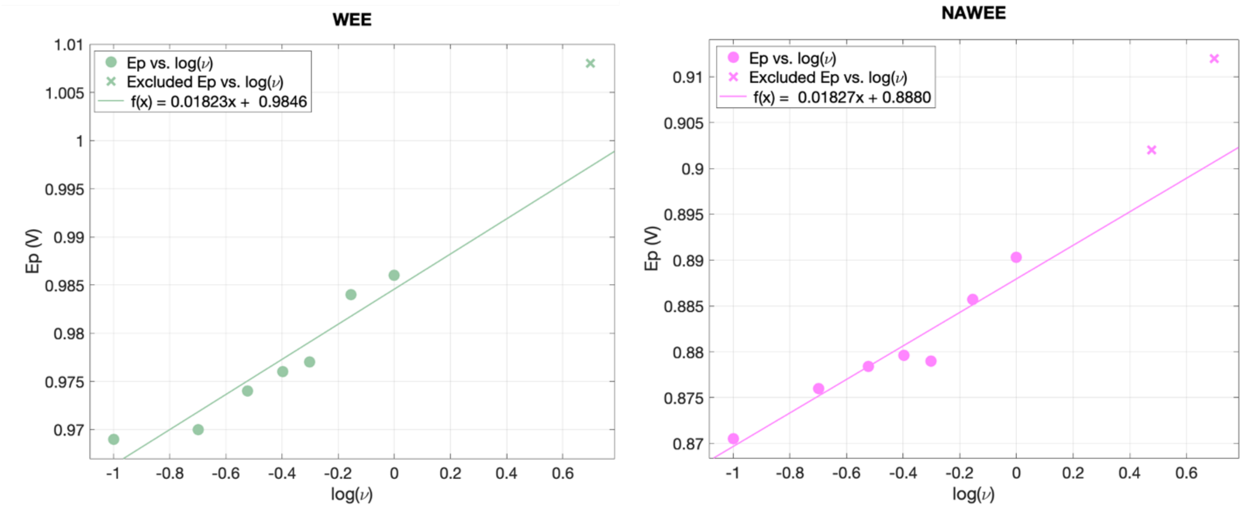

Figure S2. Peak potentials plotted as a function of log(ν) for WEE (left) and NAWEE (right). The lines represent linear fits to Eq. S1 to points having scan rates 0.1 – 1 V/s (filled circles). The data points represented by crosses were not included in the fits. Potentials are referenced to NHE.

II. Flash-Photolysis Transient Absorption of WEE

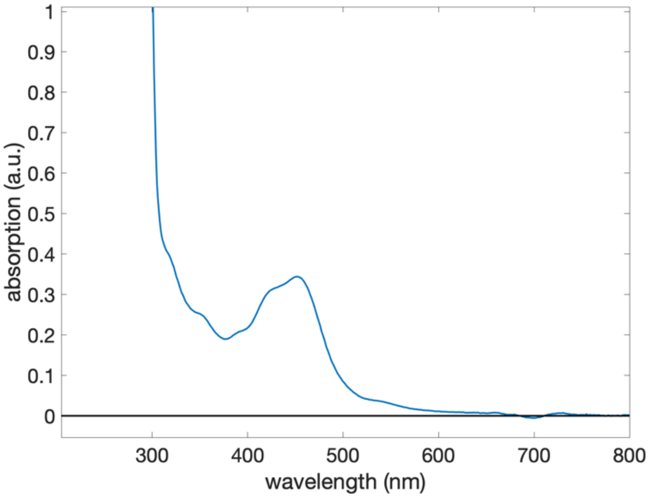

Figure S3. Steady state UV/Vis spectrum for [Ru(dmb)$_3$]$^{2+}$ ground state in deoxygenized water.

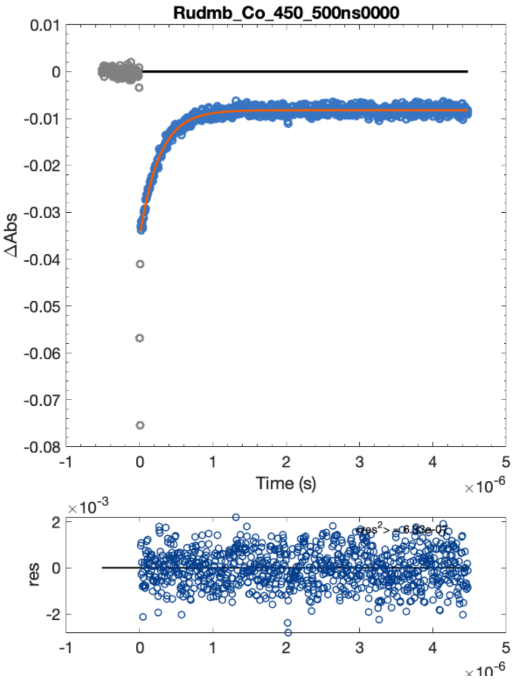

*Figure S4. Quenching of *[Ru(dmb)$_3$]$^{2+}$by 2.5 mM [Co(NH$_3$)$_5$Cl]$^{2+}$measured from the Ru(II) ground state bleach recovery at 450 nm in 0.5 mM KP$_i$ at pH = 3.6, with the fit residuals shown in the bottom graph. The single exponential fit (orange line) gave the pseudo-first order rate constant of quenching, $k_q = 3.8 \times 10^6\ s^{-1}$. Upon quenching a stable bleach (negative signal) is observed since the oxidized [Ru(dmb)$_3$]$^{3+}$does not react with anything in the solution on this timescale. The trace shows that the quenching process is finished before any reaction with WEE takes place (see traces below: Figures S6, S7 and S8).*

i. Probing the sensitivity of PCET rate constants to electrostatic changes in solution.

Formation of the encounter complex between $[Ru(dmb)_3]^{3+}$ or $[Ru(bpy)_3]^{3+}$ and WEE could be affected by changes in ionic strength at different pHs. While WEE is monocationic at pH below the pK_a of the unprotected amine-side group (pK_a = 7.5), it is neutral at pH above the pK_a. To determine how sensitive the observed rate constants are to the different electrostatic interactions between the Ru^{3+} complex and WEE, we studied the rate of WEE oxidation at three different pH values with addition of KCl to increase the ionic strength of the medium. The samples contained 200 mM KCl, 25 mM $[Ru(NH_3)_6]^{3+}$ as quencher, and 20 μM $[Ru(bpy)_3]^{2+}$ as photosensitizer. We decided to use $[Ru(bpy)_3]^{2+}$ instead of $[Ru(dmb)_3]^{2+}$ because there are no disagreements between previously published results on the values of the rate constants even at high pH using this photosensitizer. The $[Ru(NH_3)_6]^{3+}$ quencher was used instead of $[Co(NH_3)_5Cl]^{2+}$, due to the limited solubility in high concentration KCl solutions for the latter. The results are seen in Figure S5, compared to previously published data.[4] It appears that the rate constants are slightly faster at all pH values studied. A small increase in constants with a large change in ionic strength indicates that the effect of solution electrostatics has a minimal impact on PCET rates. We can therefore exclude electrostatic considerations as responsible for the observed pH dependent PCET rate constants for WEE. At pH around 10 we realize that the $[Ru(NH_3)_6]^{3+}$ quencher is less stable (it starts to undergo ligand exchange)[5-6], this is also the data point that differs the most from the previous data.[4]

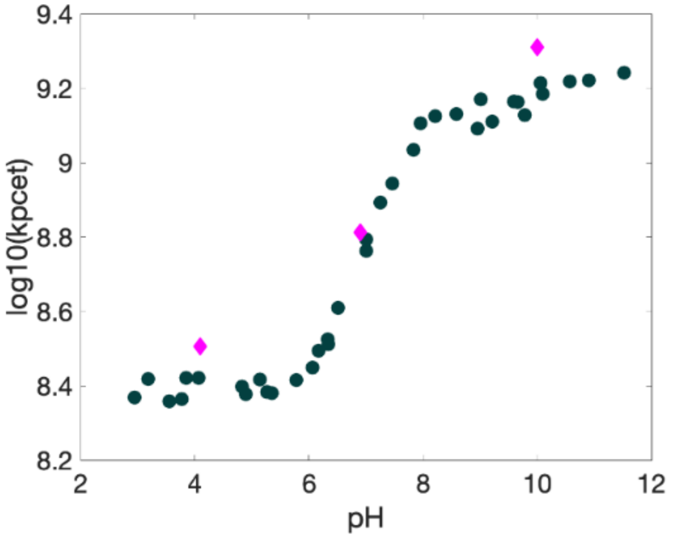

Figure S5. Logarithm of the second-order rate constants for WEE oxidation by $[Ru(bpy)_3]^{3+}$ determined as a function of pH. Black circles are from reference 4 and were collected in 0.5 mM KP_i buffer with $[Ru(bpy)_3]^{2+}$ as photosensitizer and MV^{2+} as quencher. Magenta diamonds were collected in 0.5 mM KP_i buffer with 200 mM KCl, $[Ru(bpy)_3]^{2+}$ as photosensitizer and $[Ru(NH_3)_6]^{3+}$ as quencher.

ii. TA Kinetic Traces and Rate Constants at Low pH (< 4)

The reaction kinetics was studied as a function of WEE concentration probing at 450 nm where the consumption of $[Ru(dmb)_3]^{3+}$ is monitored by the $[Ru(dmb)_3]^{2+}$ bleach recovery. At low pH (< 4) the system is near to, or below, the pK_a of oxidized tryptophan (≈ 4.3). Without deprotonation of the resulting radical, the electrochemical data in Table 1 of the main paper suggest that ΔG° ≈ +100 meV for oxidation of WEE by $[Ru(dmb)_3]^{3+}$, if the radical pK_a-value is similar to that for W and NAW. We noticed that if a sufficiently large concentration of WEE was used (> 80 mM) the rate constants appeared single exponential (Figure S7 and Table S2). Instead, at [WEE] = 50 mM and lower, the recovery did not exhibit single exponential kinetics, but showed biphasic behavior (Figure S6). The fast rate component likely represents a single ET pre-equilibrium reaction, where the pre-equilibrium position is shifted depending on the concentration of reactants. The slow component can be attributed to follow-up reactions such as PT and radical dimerization, that drive the reaction to completion. The relative amplitude of the fast (single exponential) component decreased with decreasing [WEE], and was very small for [WEE] = 5 mM (Table S1).

$$Ru^{III} + WEE \rightleftarrows Ru^{II} + WEE^{\bullet+} \quad \text{(S2)}$$

$$WEE^{\bullet+} \rightleftarrows WEE^{\bullet} + H^{+} \rightarrow \text{products} \quad \text{(S3)}$$

At [WEE] = 25 and 50 mM, the biphasic kinetics observed was well fitted by a double exponential decay. At [WEE] = 5 mM, the slow phase of the bleach recovery was fitted with a term that was second order in [$WEE^{\bullet}$]. The rate constant for the fast component had to be locked because of its small amplitude, and a value for the pseudo-first order rate constant was used that was calculated from an average of the second-order rate constant obtained from a fit to the 25 and 50 mM rate constants. We attempted to fit the 25 and 50 mM kinetic traces to single exponential plus second order recovery, however this did not converge properly, likely because the amplitude of the second phase is relatively small.

Even at the lowest concentrations of WEE employed in this pH region (5 mM), its concentration is significantly larger than the concentration of Ru^{II} on the product side ([Ru^{II}] ≈ 25 μM), so the rapid phase can be assigned to setting the equilibrium (Eq. S2), with an observed rate constant equal to the sum of the forward and backward ET rate constants. The slower component of the fits represents the further reaction in Eq. S3 that consumes both Ru^{III} and $WEE^{\bullet+}$. At [WEE] ≥ 80 mM, the equilibrium in Eq. S2 is shifted sufficiently to the right that Ru^{III} is essentially consumed already in the first phase, and the observed rate constant is close to that for the forward ET step. With [WEE] = 25 mM, about half of the Ru^{III} is consumed in each phase, seen by the similar relative amplitudes in Table S1.

At these low pH values, the protonated tryptophan radical ($WEE^{\bullet+}$) can be observed (Figure S7). Its absorption is centered at 560 nm, but it also absorbs (slightly less) at 510 nm. A comparison in

TA amplitude between the two wavelengths reveals that primarily the protonated radical is formed on the timescale of the experiment, Figure S7.[7-8]

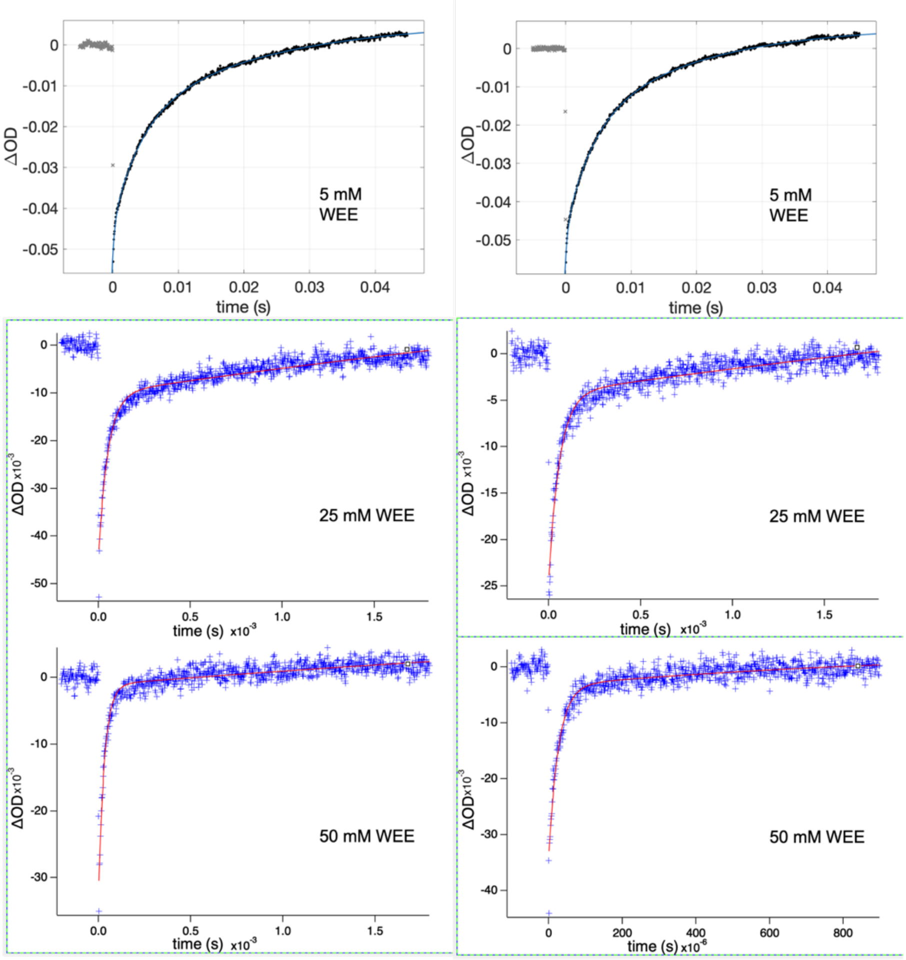

Figure S6. Representative TA traces collected at pH = 3.1(±0.1) in 0.5 mM KP$_i$ buffer. Samples contained 25 – 50 μM [Ru(dmb)$_3$]$^{2+}$and 2.5 – 5 mM [Co(NH$_3$)$_5$Cl]$^{2+}$. The traces with 5 mM WEE (top) were fit using single exponential plus second order function. The traces with 25 mM and 50 mM WEE (middle and bottom, respectively) were fit using a double exponential function. The fit parameters are given in Table S1.

Table S1. Rate constants and pre-exponential factors (with standard deviations) obtained from fits to TA traces recorded at pH = 3.1(±0.1). The values were obtained by fitting four individual single shot kinetic traces and then averaging the extracted values. For 25 mM and 50 mM WEE, a double exponential fit was used, for 5 mM WEE, a single exponential plus second order fit was used.

[WEE] (mM)	A_1 (± std)	$k_{1,\,obs}$ (± std) (s^{-1})	A_2 (± std)	$k_{2,\,obs}$ (± std) (s^{-1})
25	–2.3(±0.6)×10^{-2}	1.4(±0.1)×10^{4}	–2.8(±1.0)×10^{-2}	1.6(±0.1)×10^{2}
50	–2.3(±0.3)×10^{-2}	2.6(±0.6)×10^{4}	–1.5 (±0.9)×10^{-2}	3.3(±4) ×10^{2}
[WEE] (mM)	A_1	$k_{1,\,obs}$ (s^{-1}) (fixed)	A_2	k_2 ($M^{-1}s^{-1}$)
5	–7.7(±0.7)×10^{-3}	3.6×10^{3}	–5.5(±0.4)×10^{-2}	2.7(±0.1)×10^{3}

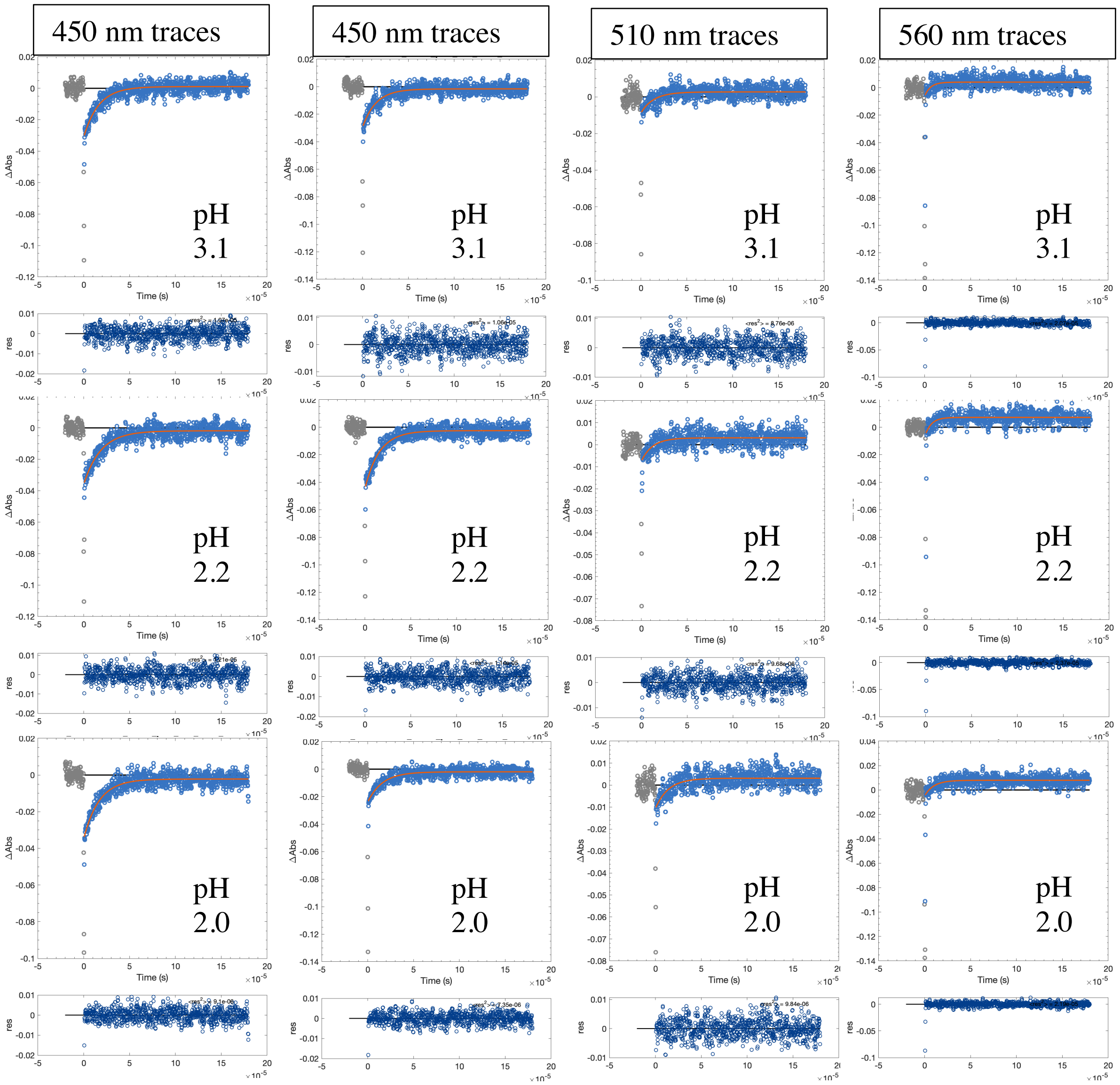

Figure S7: TA kinetic traces collected using 80 mM WEE at pH = 2 – 3 in 0.5 mM KP_i. Samples contained 25 – 50 μM $[Ru(dmb)_3]^{2+}$ and 2.5 – 5 mM $[Co(NH_3)_5Cl]^{2+}$. All traces were fit with a single exponential. The first two columns of plots correspond to 450 nm traces, the third to 510 nm traces, and the last to 560 nm traces. The top row was collected at pH = 3, the second at pH = 2.2 and the third at pH = 2.0. Fit parameters are found in Table S2.

Table S2. Rate constants (mean value and standard deviation for 5-12 traces) obtained from single exponential fits to TA traces recorded at pH = 2-3 in 0.5 mM KP_i. The rate constants shown were extracted from the 450 nm traces. The second order rate constants were obtained by dividing the first order rate constants by the concentration of WEE.

pH (±var)	[WEE] (mM)	k_{obs} (±std) (s^{-1})	k_{PCET} (±std) ($M^{-1}s^{-1}$)
2.0(±0.1)	83	6.2(±0.3)×10^4	7.5(±0.4)×10^5
2.2(±0)	81	6.6(±0.3)×10^4	8.1(±0.4)×10^5
3.1(±0.1)	84	6.1(±0.5)×10^4	7.3(±0.6)×10^5

iii. TA Kinetic Traces and Rate Constants at Intermediate pH (5 < pH < 7)

Figure S8 show the transient absorption (TA) single shot kinetic traces with single exponential fits (orange) used to extract the rate constants seen in Figure 5, main text. Below each TA trace is the fit residuals. Traces were collected on two separate days with independently prepared samples containing different concentrations of WEE, $[Ru(dmb)_3]^{2+}$ and $[Co(NH_3)_5Cl]^{2+}$. Rate constants obtained from fits to 450 nm traces are shown in Table S3.

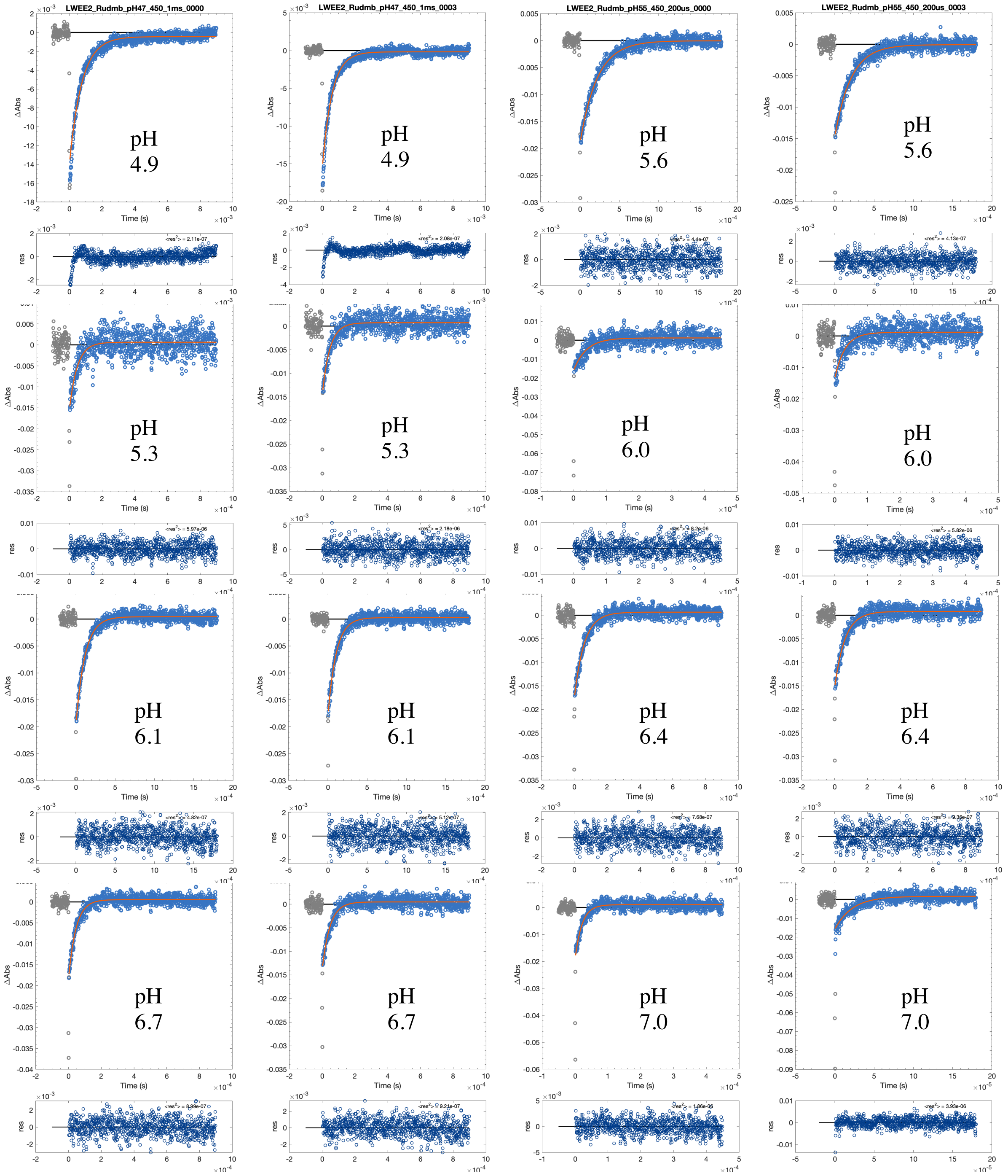

Figure S8. TA kinetic traces collected at pH = 4.7 – 7.0 in 0.5 mM KP$_i$. Samples contained 25 – 50 µM [Ru(dmb)$_3$]$^{2+}$, 2.5 – 5 mM [Co(NH$_3$)$_5$Cl]$^{2+}$ and 2.3 – 9.9 mM NAWEE. Fit values are shown in Table S3. All traces were collected at 450 nm. The orange line in each graph represents a single exponential fit. The fit residuals are seen below the TA trace.

Table S3. Rate constants determined for WEE oxidation at pH = 4.9–7.0 in 0.5 mM KP_i (mean value and standard deviation for 4-8 traces at each pH-value).

pH (±var)	[WEE] (mM)	k_{obs} (±std) (s^{-1})	k_{PCET} (±std) ($M^{-1}s^{-1}$)
4.9(±0.2)	2.3	1.5(±0.4)×10^3	6.4(±2)×10^5
5.3(±0.1)	9.9	2.0(±0.2)×10^4	2.0(±0.2)×10^6
6.0(±0.1)	9.9	2.9(±0.3)×10^4	3.0(±0.3)×10^6
5.6(±0.1)	2.3	4.6(±0.2)×10^3	2.0(±0.1)×10^6
6.1(±0.1)	2.3	9.5(±0.6)×10^3	4.2(±0.2)×10^6
6.45(±0.1)	2.3	1.6(±0.1)×10^4	7.1(±0.4)×10^6
6.7(±0.1)	2.3	2.2(±0.1)×10^4	9.6(±0.4)×10^6
7.05(±0.1)	2.3	4.7(±0.1)×10^4	2.0(±0.04)×10^7

iv. TA Kinetic Traces and Rate Constants at High pH (> 7)

Excitation of $[Ru(dmb)_3]^{2+}$ with a 355 nm 50 mJ laser pulse results in two-photon ionization which produces Ru(III) and solvated electrons (e^-_{solv}). There are several species that can absorb at the wavelengths where we typically monitor the WEE oxidation (450 nm for Ru(II) bleach and 510 nm for $WEE^•$ formation), extinction coefficients and reaction steps with difference extinction coefficients for each reaction, and rate constants are found in Table S4 and Scheme S1, respectively. The e^-_{solv} exhibits a broad absorption band centered around 700 nm that extends towards the blue part of the spectrum.[9] The scheme does not include reaction between e^-_{solv} and $WEE^•$ because the solvated electrons were found to decay before $WEE^•$ was formed (vide infra).

Table S4. Extinction coefficients at 450 and 510 nm for the species present in a solution containing $[Ru(dmb)_3]^{2+}$ and WEE following two-photon ionization of $[Ru(dmb)_3]^{2+}$.

Species	ε(450 nm) / $M^{-1}cm^{-1}$	ε(510 nm) / $M^{-1}cm^{-1}$
Ru(II)	14 800[10]	3 100[10]
Ru(III)	2 300[10]	-
Ru(II)(L^-)	10 700[10]	9 800[10]
e^-_{solv}	4500	8 000[9]
$WEE^•$	-	2 300[11]

Scheme S1. Reaction steps following two-photon ionization of $[Ru(dmb)_3]^{2+}$ in the presence of WEE, their expected effect on the transient absorption signals at 450 nm and 510 nm (approximate difference extinction coefficients, Δε, for the reaction shown), and rate constants.

		Δε / $M^{-1}cm^{-1}$		k / $M^{-1}s^{-1}$ [a]
		450 nm	510 nm	
(1)	Ru(III) + e^-_{solv} → Ru(II)	+8 000	–4 900	5.2×10^{10}
(2)	Ru(III) + WEE → Ru(II) + $WEE^•$ + H^+	+12 500	+5 400	2.0×10^{6} [b]
(3)	Ru(II) + e^-_{solv} → Ru(II)(L^-)	–8 600	–1 300	5.2×10^{10}
(4)	Ru(III) + Ru(II)(L^-) → 2 Ru(II)	+16 600	–3 600	3.4×10^{9}
(5)	$WEE^•$ + Ru(II)(L^-) + H^+ → WEE + Ru(II)	+4 100	–9 000	1.5×10^{8} [c]

[a]*From ref 12, unless otherwise indicated.* [b]*From Table S3 at pH = 7.* [c]*See text.*

Upon two-photon ionization, Ru(III) and e^-_{solv} are formed. The e^-_{solv} can decay via (1) or (3), both of which are very fast, diffusion controlled, reactions.[12] Since [Ru(II)] > [Ru(III)], reaction (3) is expected to dominate. This is indeed what is observed in Figure S9, where the electron signal (broad absorption around 700 nm), decays on the time scale of 200 – 1000 μs as Ru(II)L^- is formed (band maximum around 510 nm). The 450 nm Ru(II) bleach recovery shows a biphasic behavior, with a strong ~300 ns component that is followed by a much slower recovery on the time scale of 10 μs (Figure S15). The fast component is due to decay of the *Ru(II) that did not absorb a second photon, but the remaining bleach in the slow component shows that ca. 1 μM Ru(III) is formed. Kinetic traces recorded at 450 nm and 540 nm with only $[Ru(dmb)_3]^{2+}$ in 0.5 mM KP_i indicate that the 450 nm bleach recovery occurs with the same rate constant as for the Ru(II)L^- decay, $k \approx 7\times10^3$ s^{-1} from a fit to a first order recovery at 540 nm and 450 nm, Figure S10. This indicates that without WEE in solution, Ru(II)L^- mainly decay via recombination with Ru(III). One would expect the decay to exhibit second order kinetics, however such a fit did not converge, probably because reaction (4) is not completely dominating. The first order fits presented below are not perfect, but can be used to indicate similarities or differences between decays at different wavelengths and pH-values. When 0.1 mM WEE is added to the solution, the 450 nm recovery is accelerated and the decays fit a single exponential.

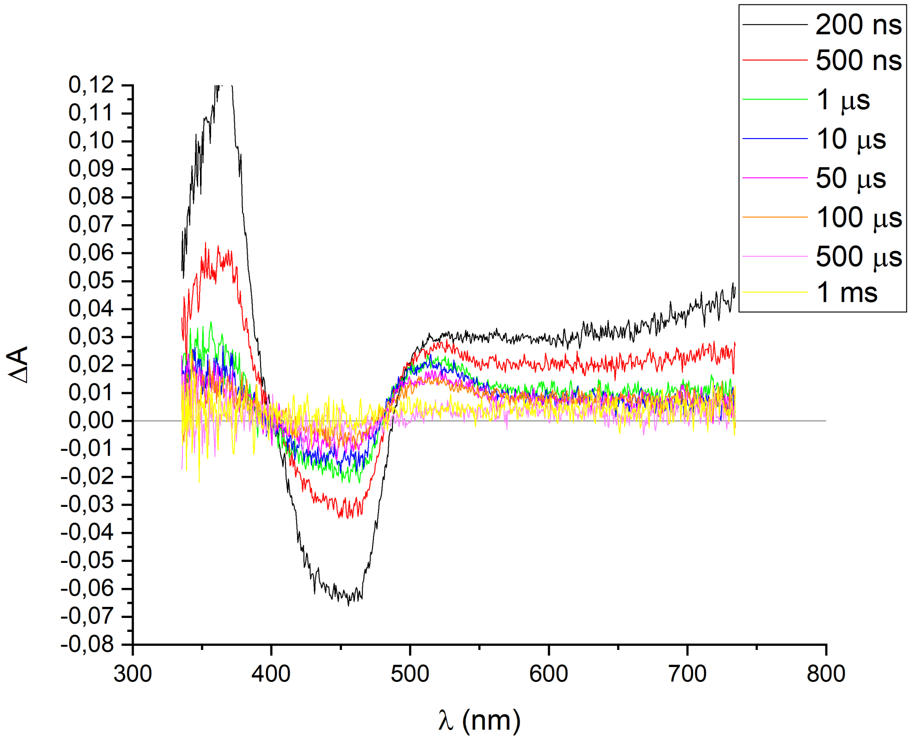

*Figure S9. TA Spectra of $[Ru(dmb)_3]^{2+}$ following two-photon ionization by 355 nm laser pulse. Sample contained 20 µM $[Ru(dmb)_3]^{2+}$ in 0.5 mM KP_i buffer at pH = 7.5. Delay times are seen in the caption. The spectra have been corrected for *Ru(II) fluorescence.*

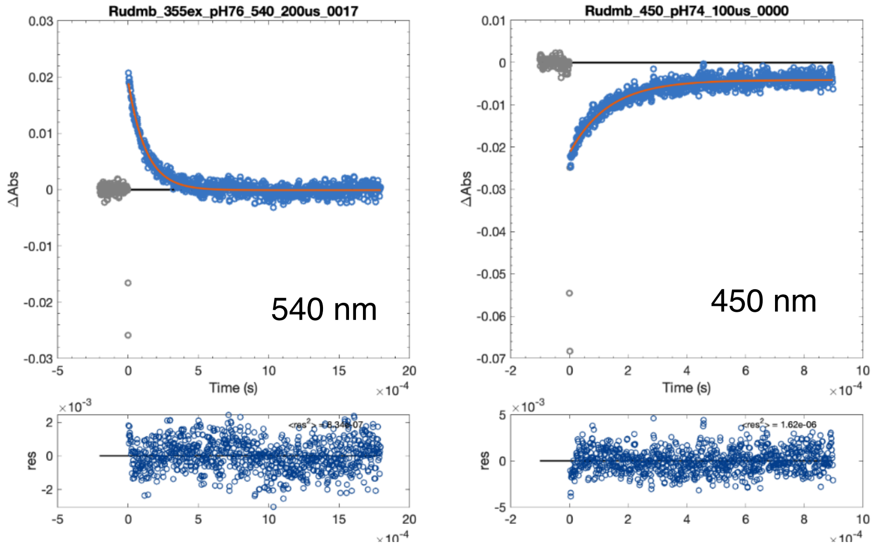

Figure S10. TA kinetic trace at 540 nm (left) at pH = 7.6, and 450 nm (right), following two-photon ionization by 355 nm laser pulse of a sample containing only $[Ru(dmb)_3]^{2+}$ in 0.5 mM KP_i at pH = 7.4. The orange line represents a single exponential fit, with the fit residuals shown in the bottom graph.

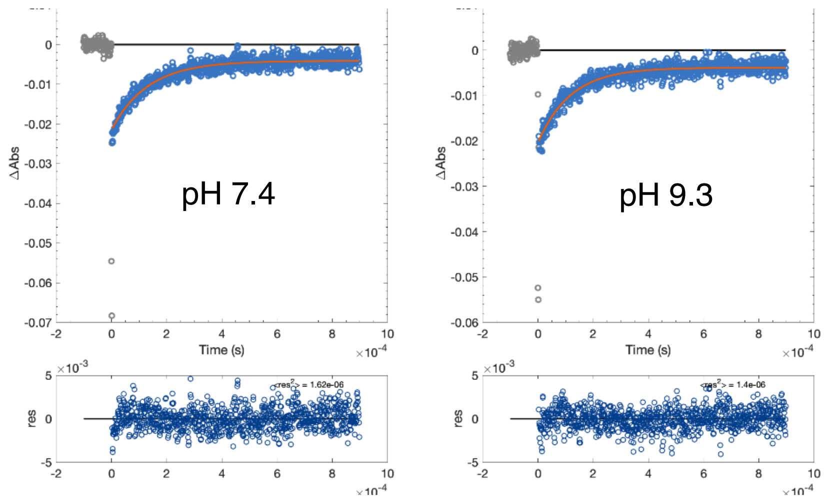

Figure S11. TA kinetic trace at 450 nm following two-photon ionization by a 355 nm laser pulse of a sample containing only [Ru(dmb)$_3$]$^{2+}$ in 0.5 mM KP$_i$, at pH = 7.4 (left) and pH = 9.3 (right). The orange line represents a single exponential fit, with the fit residuals shown in the bottom graph. The traces show that recombination between Ru(III) and Ru(II)L$^-$ does not exhibit any pH-dependence.

In the presence of WEE, the 450 nm recovery is accelerated, and exhibits first order kinetics with $k = 1.4\times10^4$ s^{-1} at pH = 7.6; Figure S12 and Table S5 show traces and data for all pH values studied using this method. The rate constant should represent the sum of the rate constants for reactions (2) and (4). It is clear that the 450 nm recovery becomes faster as the pH increases. Subtracting the rate constant for 450 nm recovery without WEE (reaction (4) in Scheme 1) from the rate constants with WEE gives a corrected rate constant (k_{corr}) which represents reaction (2) in Scheme S1. These are the rate constants found in Figure 5 in the main paper.

The rate of 450 nm bleach recovery as a function of pH in the absence of WEE was also determined. The kinetic traces, seen in Figure S11, show that this recovery is independent of pH, showing that the pH-dependent rate constants are due to reaction with WEE.

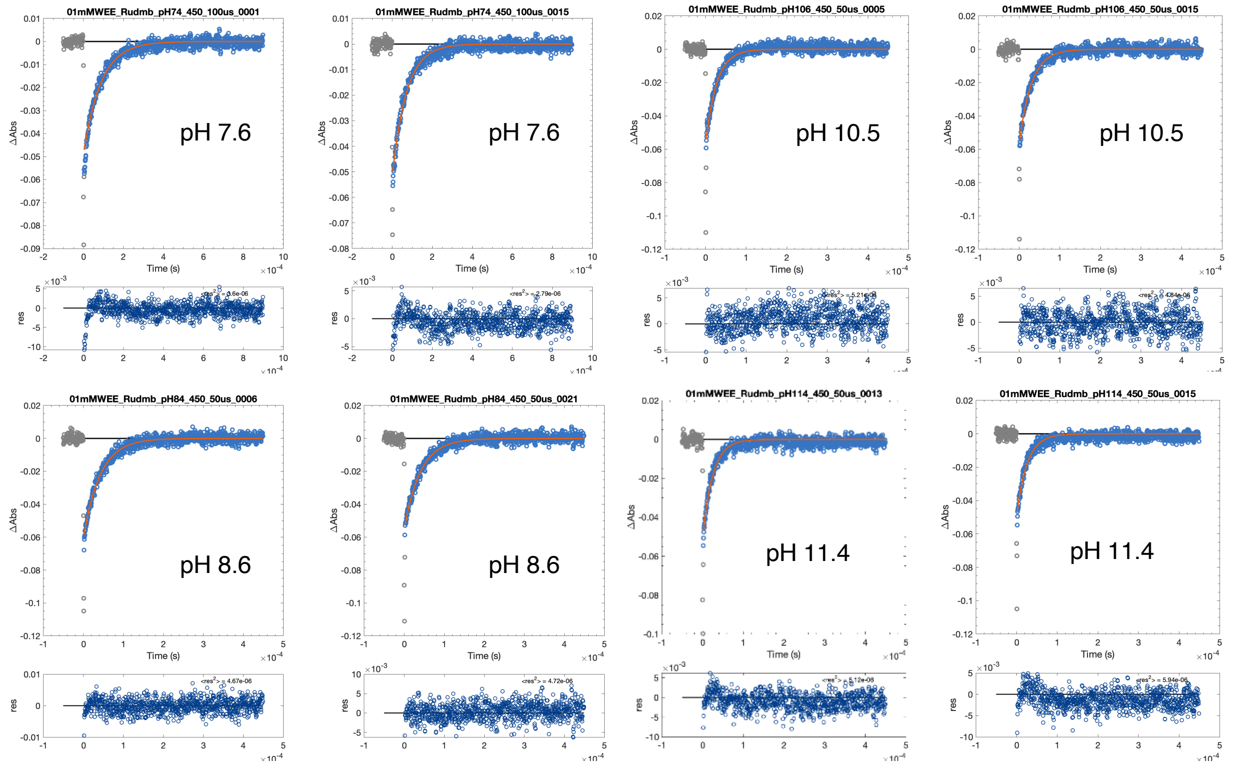

Figure S12. TA kinetic traces at 450 nm following two-photon ionization by 355 nm laser pulse of 20 μM $[Ru(dmb)_3]^{2+}$ and 100 μM WEE in 0.5 mM KP_i, at pH = 7.6 – 11.4. Orange lines represent single exponential fits, obtained rate constants are found in Table S5. Note that the traces at pH = 7.6 use a time window of 1 ms, while the other traces use 500 μs.

Table S5. Rate constants determined at high pH following two-photon ionization forming $[Ru(dmb)_3]^{3+}$, k_{PCET} was calculated by dividing the corrected k_{obs} value by the concentration of WEE used. The values in the final column are plotted in Figure 5, main text.

pH (±var)	k_{obs} (s^{-1})	$k_{Ru(II)L\text{-}}$ recovery(s^{-1})	$k_{corrected}$ (s^{-1})	k_{PCET}, corrected ($M^{-1}s^{-1}$)
7.6 (±0.2)	1.4×10^4	7×10^3	6.4×10^3	6.4×10^7
8.6 (±0.3)	2.3×10^4	7×10^3	1.6×10^4	1.6×10^8
10.5 (±0.1)	3.5×10^4	7×10^3	2.8×10^4	2.8×10^8
11.4 (±0.1)	4.3×10^4	7×10^3	3.5×10^4	3.5×10^8

When WEE is present in the solution, the observed rate constants determined at 510 nm (which are dominated by $Ru(II)L^-$ decay) is obtained as ~1.5×10^4 s^{-1} (Figure S13 right panel), and the kinetics is independent of pH. This is slower than the disappearance of Ru(III), which shows that another oxidized species is formed to balance the electrons on $Ru(II)L^-$, which is attributed to $WEE^{•}$. Some absorption around 450 nm from $WEE^{•}$ can also explain why the 450 nm traces return to baseline, (Figure S13, left panel) even though $Ru(II)L^-$ shows a bleach at this wavelength. The

WEE$^{\bullet}$ then decays via dimerization, $k_{dim} \approx 8{\times}10^{8}$ $s^{-1}M^{-1}$,[13] or recombination with Ru(II)L-, with a rate constant that seems to be similar to that for reaction (3), i.e. $k \approx 3{\times}10^{9}$ $s^{-1}M^{-1}$.
or reaction with Ru(II)L^{-}, $k \approx 1.5{\times}10^{4}$ s^{-1} at 0.1 mM WEE, which gives a second-order rate constant $\approx 1.5{\times}10^{8}$ $M^{-1}s^{-1}$.

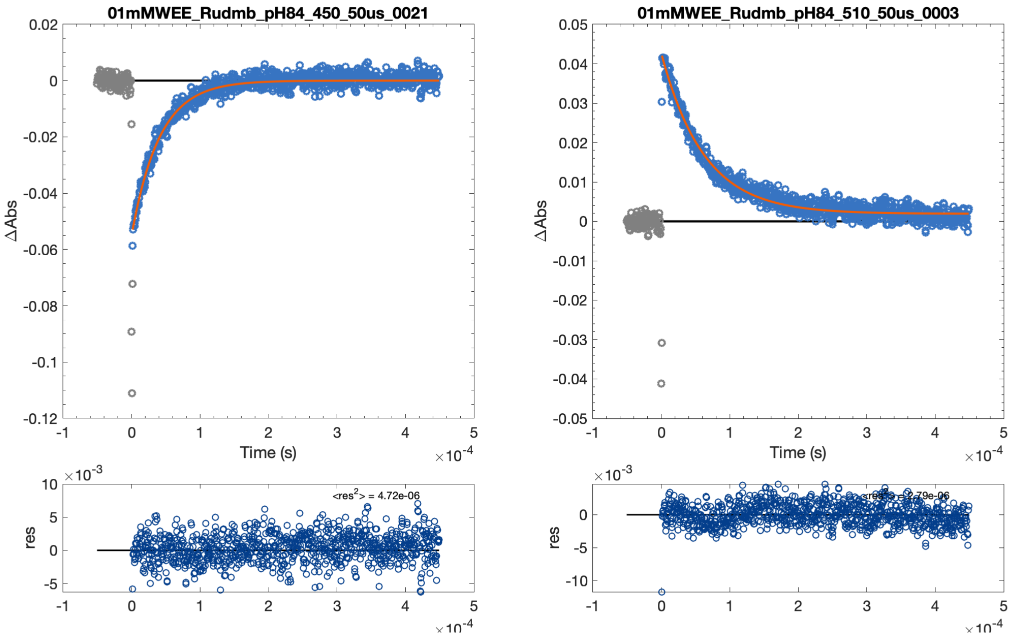

Figure S13. TA kinetic trace at 450 nm (left) and 510 nm (right) following two-photon ionization by 355 nm laser pulse of a sample containing $[Ru(dmb)_3]^{2+}$ and 0.1 mM WEE in 0.5 mM KP_i, at pH = 8.6 . The orange line represents a single exponential fit, with the fit residuals shown in the bottom graph.

To ensure that the 450 nm recovery was first order with respect to [WEE], the rate using 1 mM WEE at pH 8.8 was measured, Figure S14. The bleach recovery exhibited double exponential recovery with $k_1 = 3.0{\times}10^{6}$ s^{-1} (*Ru(II) decay) and $k_2 = 1.5{\times}10^{5}$ s^{-1} (PCET with WEE). The slow component agrees well with the recovery using 0.1 mM WEE in pH 8.6 (k = $1.6{\times}10^{8}$ $M^{-1}s^{-1}$, and $1.6{\times}10^{8}$ $M^{-1}s^{-1}$, for 0.1 mM and 1 mM WEE, respectively). The very similar second order rate constants obtained using 0.1 mM and 1 mM WEE confirms that the reaction is first order in [WEE].

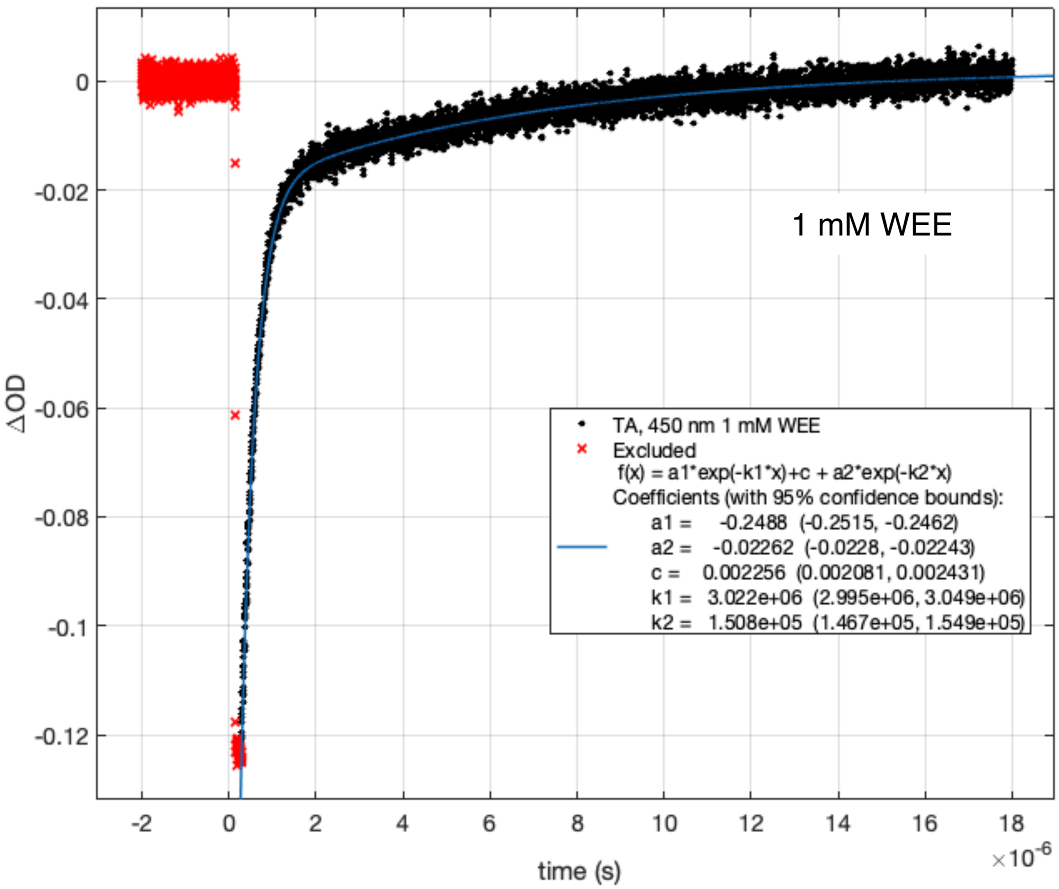

Figure S14. TA kinetic trace at 450 nm following two-photon ionization by 355 nm laser pulse of 20 μM [Ru(dmb)$_3$]$^{2+}$ and 1 mM WEE in 0.5 mM KP$_i$ at pH = 8.8. The blue line represents a fit to a double exponential function. Fit parameters seen in the figure caption.

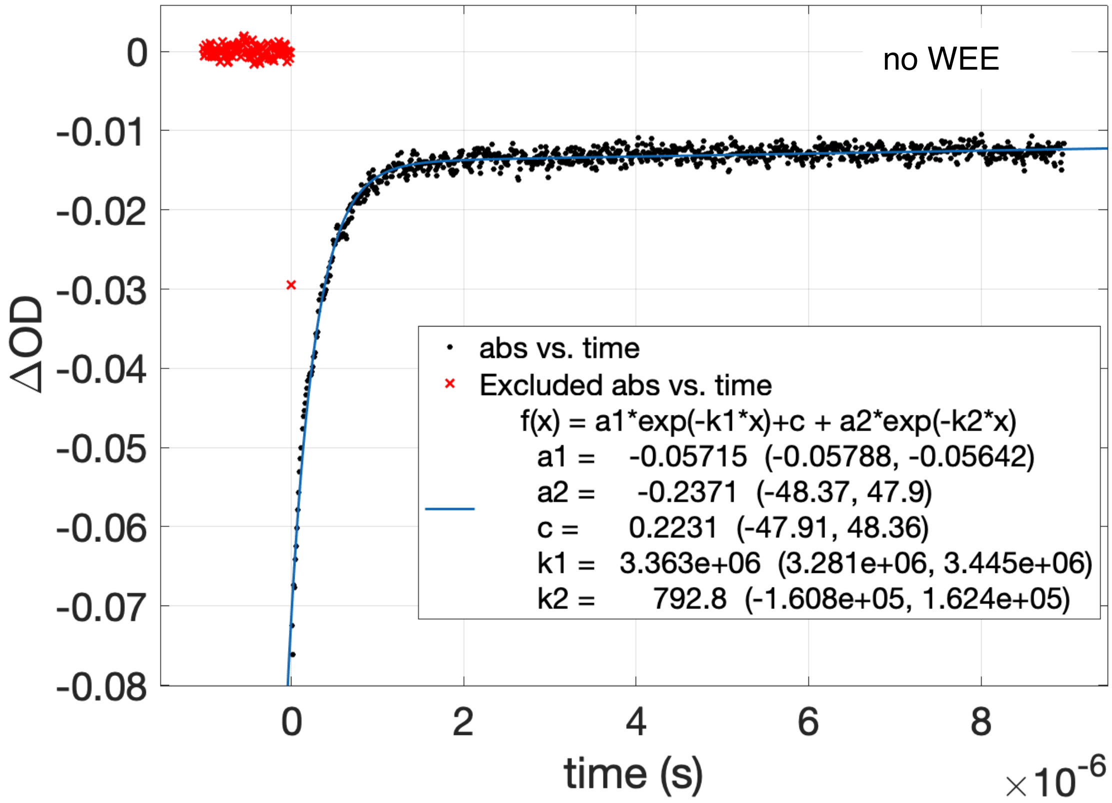

Figure S15. TA kinetic trace at 450 nm following two-photon ionization by 355 nm laser pulse of 20 μM [Ru(dmb)$_3$]$^{2+}$ in 0.5 mM KP$_i$ at pH 7.5. The blue line represents a fit to a double exponential function. Fit parameters seen in the figure caption.

III. Flash-Photolysis Transient Absorption of NAWEE

i. NAWEE oxidation with $[Ru(dmb)_3]^{2+}$ as photosensitizer

Rate constants for NAWEE oxidation by $[Ru(dmb)_3]^{3+}$ were measured as a function of pH. $[Ru(dmb)_3]^{3+}$ was generated with $[Co(NH_3)_5Cl]^{2+}$ as an irreversible quencher. The rate constants of NAWEE oxidation exhibited no dependence on pH and are in good agreement with previously published data for NAWEE using the same photosensitizer but with $[Ru(NH_3)_6]^{3+}$ as the quencher,[6] as seen in Figure S16. The pH independent rate constants determined with $[Ru(dmb)_3]^{3+}$ and $[Ru(NH_3)_6]^{3+}$ as the photosensitizer and quencher, respectively, are indicative of an ET limited reaction, i.e. an ETPT mechanism.

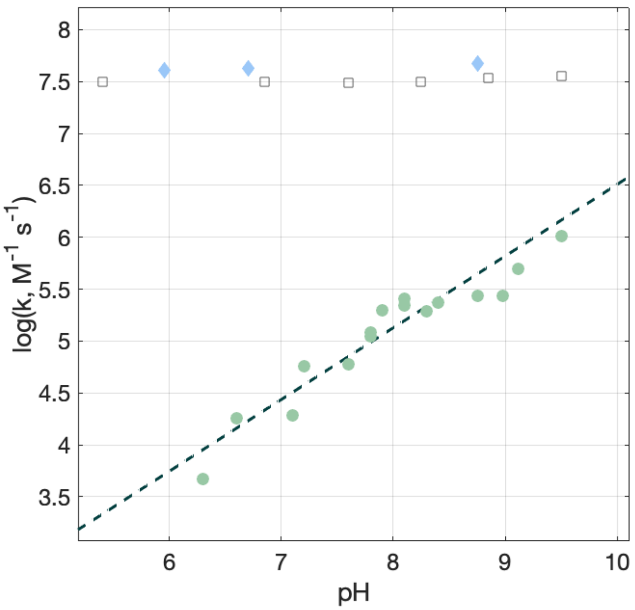

Figure S16. Rate constants for NAWEE oxidation as a function of pH. Green filled circles: 0.3 – 0.4 mM NAWEE, 20 – 25 µM $[ZnTPPS]^{3-}$ as photosensitizer and 2.5 – 5 mM $[Co(NH_3)_5Cl]^{2+}$ as quencher. Filled blue diamonds: 1 mM NAWEE, 25 – 50 µM $[Ru(dmb)_3]^{3+}$ as photosensitizer and 2.5 – 5.0 mM $[Co(NH_3)_5Cl]^{2+}$ as quencher. Unfilled gray squares: NAWEE with $[Ru(dmb)_3]^{3+}$ as photosensitizer and $[Ru(NH_3)_6]^{3+}$ as quencher, from ref. 6.

ii. Identifying Rate Constants Associated with NAWEE Oxidation by $[ZnTPPS]^{4-}$

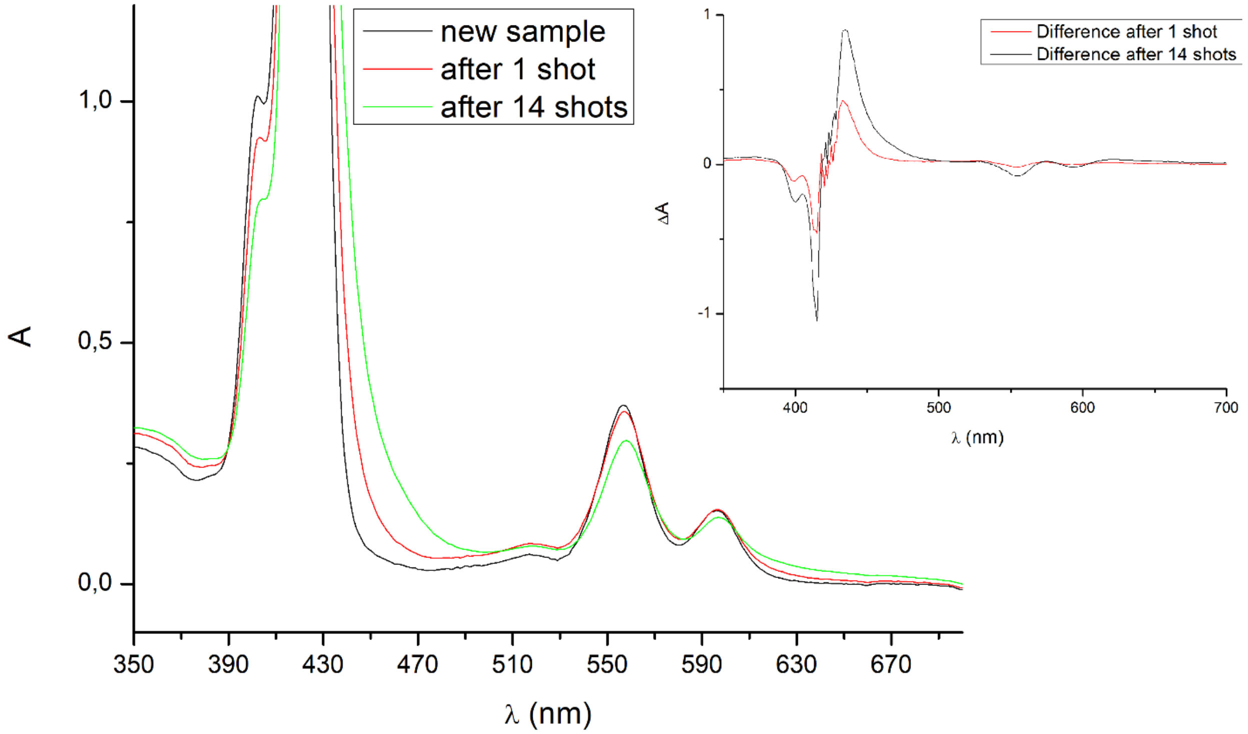

Figure S17. Comparison between UV-Vis spectra of a ZnTPPS sample before and after TA spectroscopy. Sample contained 0.5 mM KP_i, 17μM ZnTPPS, 0.50 mM $[Co(NH_3)_5Cl]^{2+}$, and 0.31 mM NAWEE. The inset shows the difference spectra after 1 and 14 shots.

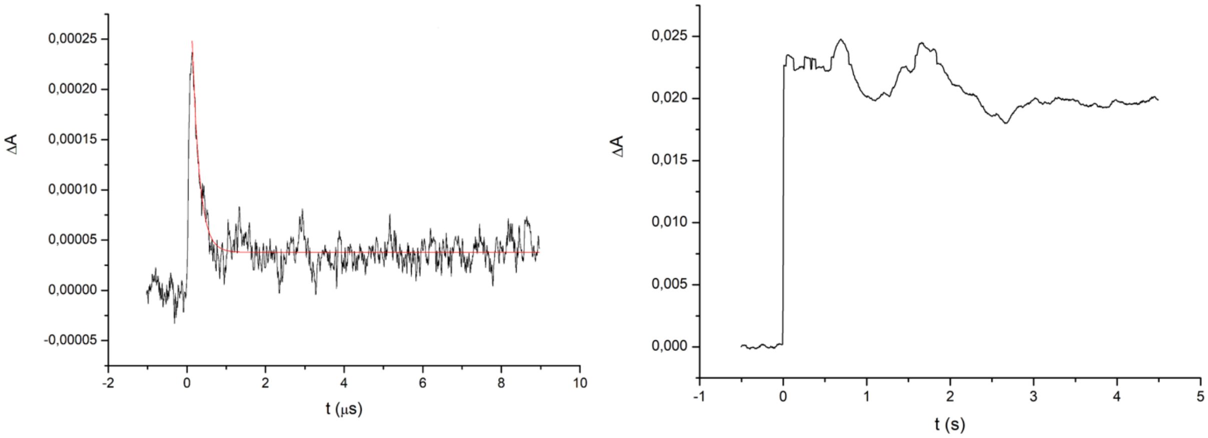

Figure S18: TA traces measured at 470 nm at a pH value of 6.6. Sample contained 0.5 mM KP_i, 20 μM $[ZnTTPS]^{4-}$ and 1.2 mM $[Co(NH_3)_5Cl]^{2+}$. Left figure includes a single exponential fit which gave the rate of quenching = $4.9{\times}10^6$ s^{-1} under the present conditions. Right figure shows stability of the (oxidized) $[ZnTTPS]^{3-}$ species over 5 seconds. The changes in intensity are due to lamp instability.

$[ZnTPPS]^{4-}$ was excited in the Q-band (545 nm) and spectral changes were studied at 470 nm, in the absorption band of the oxidized form of the porphyrin. The kinetic traces recorded at 470 nm in the presence of NAWEE fit to a double exponential function that did not return to the baseline.

Upon laser flash-photolysis, the steady state UV-Vis spectrum of $[ZnTPPS]^{4-}$ changed, showing increased intensity between 440 – 470 nm, Figure S17. This indicated that $[ZnTPPS]^{4-}$ degrades during the TA experiment, the products of which absorbed at 470 nm. For this reason, all kinetic traces used are from the first laser shot to a fresh sample. The difference spectra in the inset of Figure S17 show the changes in the UV-Vis spectra after degradation.

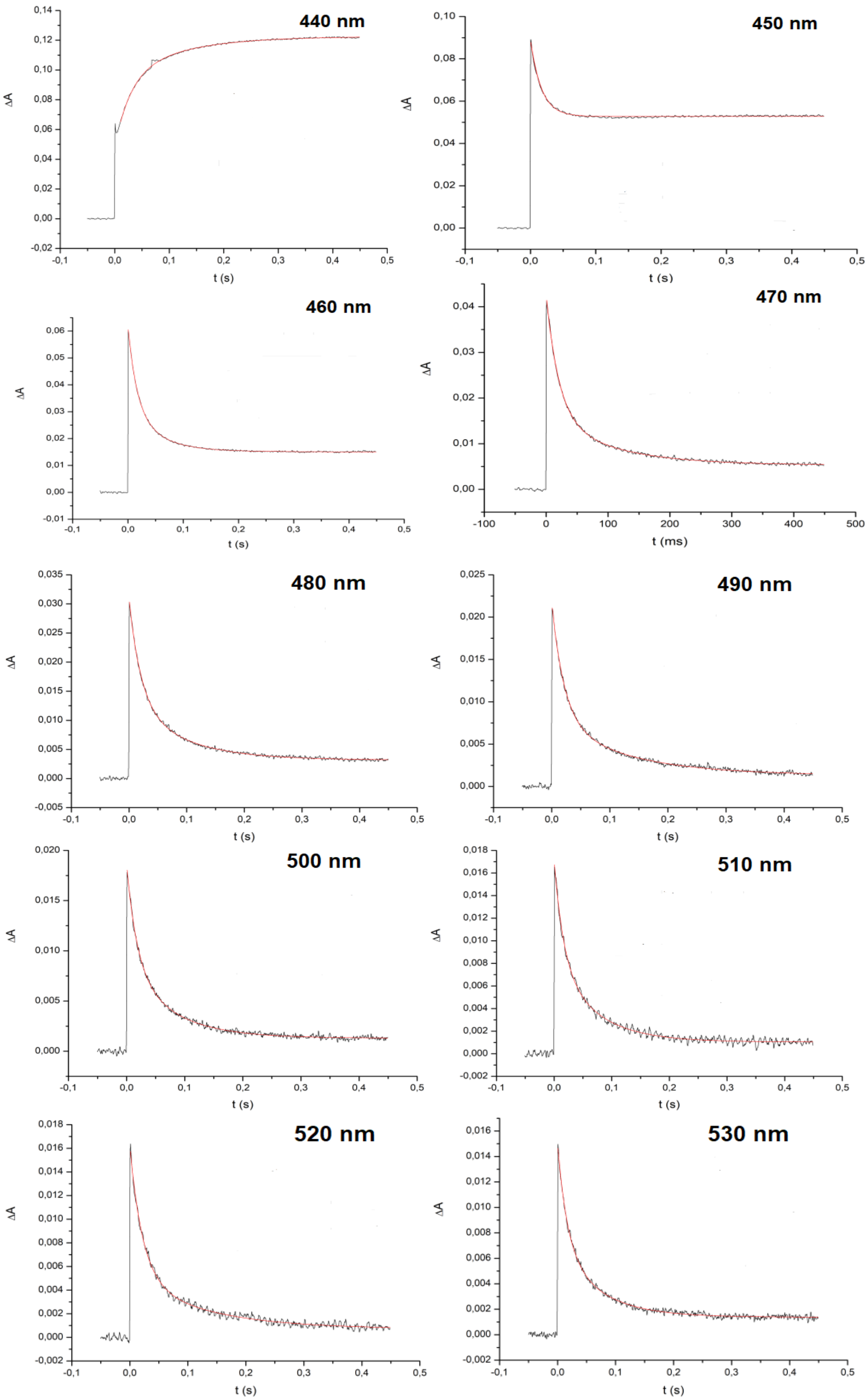

Figure S19:TA traces measured at different wavelengths from 440 nm to 530 nm to build transient spectra. Sample contained 0.5 mM KP_i, 20 μM $[ZnTTPS]^{4-}$, 0.53 mM $[Co(NH_3)_5Cl]^{2+}$ and 0.42 mM NAWEE. Fit parameters are shown in Table S6.

Table S6. Rate constants, pre-exponential factors and baseline offset (Y_0) obtained from a double exponential fit to TA traces measured at different probe wavelengths, seen in Figure S19.

Probe λ (nm)	pH (±var)	Y0	A_1	$k_{1,PCET}$ ($M^{-1}s^{-1}$)	A_2	$k_{2,PCET}$ ($M^{-1}s^{-1}$)
440	7.7 (±0.1)	0.12	-0.045	8.4×10^4	-0.029	2.1×10^4
450[a]	7.8 (±0.2)	5.3×10^{-2}	0.036	1.4×10^5	-	-
460	7.8 (±0.1)	1.5×10^{-2}	0.031	1.4×10^5	0.016	4.4×10^4
470	7.8 (±0.2)	5.3×10^{-3}	0.026	1.2×10^5	0.011	2.2×10^4
480	7.8 (±0.2)	3.2×10^{-3}	0.018	1.2×10^5	0.010	2.6×10^4
490	7.8 (±0.2)	1.3×10^{-3}	0.014	1.1×10^5	0.0066	1.9×10^4
500	7.7 (±0.2)	1.3×10^{-3}	0.010	1.3×10^5	0.0072	3.1×10^4
510	7.7 (±0.2)	1.0×10^{-3}	0.0088	1.4×10^5	0.0072	3.4×10^4
520	7.7 (±0.2)	7.0×10^{-4}	0.011	1.1×10^5	0.0044	1.9×10^4
530	7.7 (±0.2)	1.4×10^{-3}	0,0079	1.4×10^5	0.0057	3.4×10^4

[a]*Fit using a single exponential function.*

iii. TA Kinetic Traces Used to Determine the pH Dependence of NAWEE Oxidation

NAWEE oxidation kinetics were extracted from 470 nm kinetic traces. Double exponential fits to 470 nm traces are shown in Figure S20 and Table S7 at pH 6.3 – 9.5.

Using a weaker oxidant led to slower kinetics, and NAWEE had limited solubility in water that hindered us from collecting faster observed pseudo-first order rate constants. Therefore, kinetic traces for this system were collected on very long timescales up to 10 s. Extra precautions were taken to accommodate collection of TA traces on long timescales. Signals settings were optimized using a dummy sample so that each kinetic trace was collected on a fresh sample that had not been exposed to light. Exposure to excess probe light was limited during detection by filtering the probe light through a monochromator such that FWHM was 18.6 nm before the sample. Taking this step ensures minimal initiation of the photosensitizer/quencher reaction which accelerate $[ZnTTPS]^{4-}$ degradation. Xe lamps fluctuate in intensity on longer timescales. This unavoidable effect can be seen in traces collected on longer timescales in Figure S20. To minimize random fluctuations of the Xe lamp, the temperature was equilibrated for at least 30 minutes up prior to measurements. Importantly, the fluctuations are random and good exponential fits are still obtained.

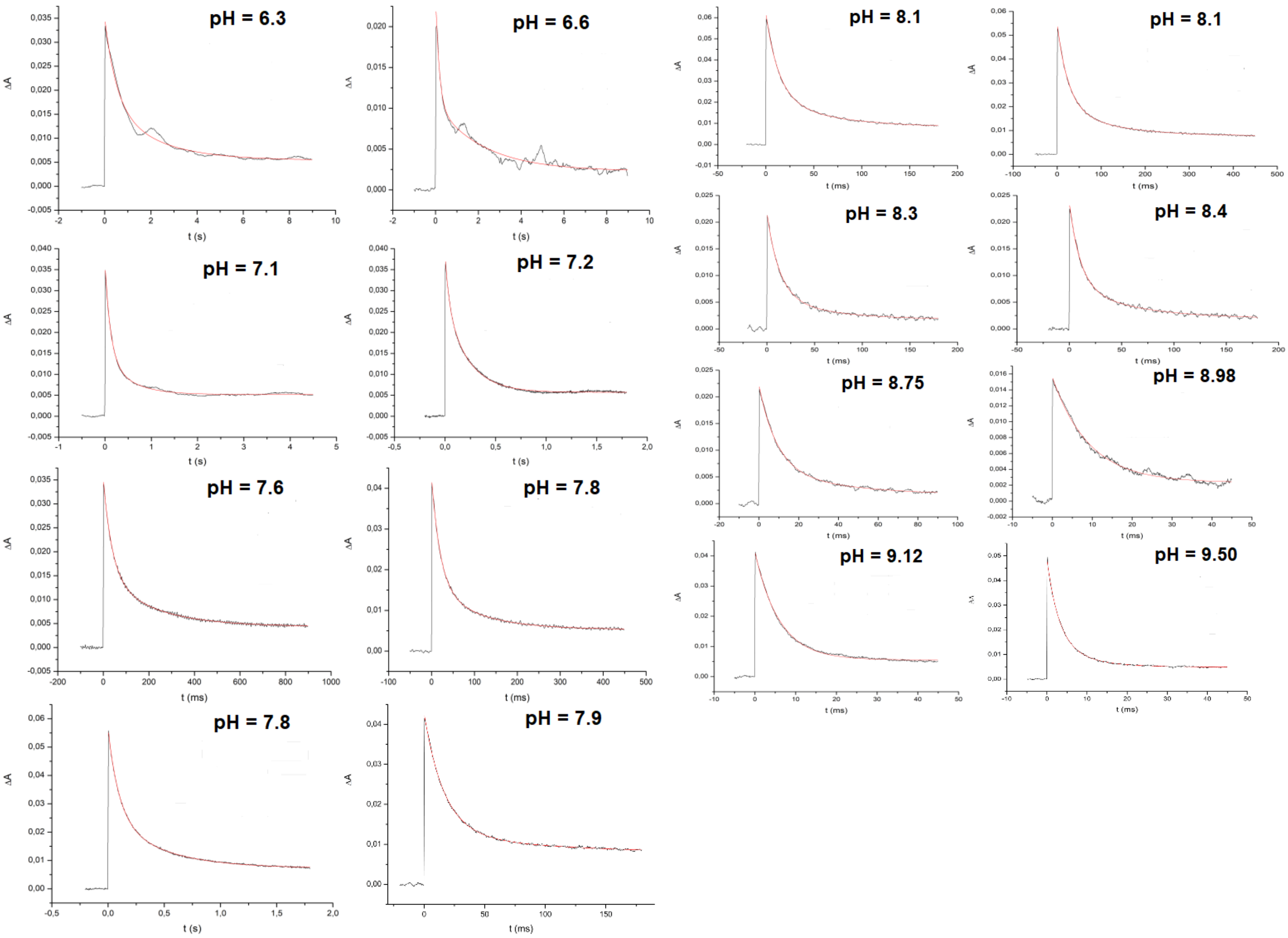

Figure S20. Single laser shot TA traces measured at 470 nm at different pH values from 6.3 to 9.5. Sample contained 0.5 mM KP_i, 15 – 25 μM $[ZnTTPS]^{4-}$, 0.4 – 2 mM $[Co(NH_3)_5Cl]^{2+}$ and 0.3 – 0.4 mM NAWEE. Fit parameters are shown in Table S7.

Table S7: Rate constants, pre-exponential factors and baseline offset (Y_0) obtained from a double exponential fit to TA traces used to calculate pH dependence. Sample contained $[ZnTPPS]^{3-}$, $[Co(NH_3)_5Cl]^{2+}$ and NAWEE.

pH (±var)	Y_0	A_1	k_1/[NAWEE] ($M^{-1}s^{-1}$)	A_2	k_2/[NAWEE] ($M^{-1}s^{-1}$)
6.3 (±0.3)	0.0054	0.017	4.7×10^{3}	0.012	1.2×10^{3}
6.6 (±0.2)	0.0023	0.013	1.8×10^{4}	0.0082	1.4×10^{3}
7.1 (±0.2)	0.0052	0.022	1.9×10^{4}	0.0089	4.5×10^{3}
7.2 (±0.2)	0.0057	0.011	5.7×10^{4}	0.022	1.1×10^{4}
7.6 (±0.2)	0.0044	0.020	6.0×10^{4}	0.011	1.1×10^{4}
7.8 (±0.2)	0.0053	0.026	1.2×10^{5}	0.011	2.2×10^{4}
7.8 (±0.2)	0.0072	0.031	1.1×10^{5}	0.018	2.6×10^{4}
7.9 (±0.2)	0.0077	0.030	2.0×10^{5}	0.0052	3.2×10^{4}
8.1 (±0.3)	0.0081	0.039	2.6×10^{5}	0.015	5.5×10^{4}
8.1 (±0.2)	0.0077	0.034	2.2×10^{5}	0.012	4.7×10^{4}
8.3 (±0.2)	0.0017	0.015	1.9×10^{5}	0.0047	4.5×10^{4}
8.4 (±0.2)	0.0019	0.015	2.4×10^{5}	0.0063	4.3×10^{4}
8.75 (±0.04)	0.0015	0.016	2.8×10^{5}	0.0049	6.2×10^{4}
8.98 (±0.01)[a]	0.0023	0.013	2.7×10^{5}	-	-
9.12 (±0.04)[a]	0.0056	0.036	5.0×10^{5}	-	-
9.50 (±0.08)	0.0048	0.033	1.0×10^{6}	0.011	3.6×10^{5}

[a]Fit using a single exponential function.

iv. Rate Constants for NAWEE Oxidation as a Function of Buffer Concentration

TA traces have been measured in samples containing different concentrations of buffer from 0.5 to 5 mM. Rate constants have been calculated from the short component of a double exponential fit to assess the contribution of the buffer as an alternative proton acceptor in the PCET reaction. The results are shown in Table S8. One order of magnitude increase in buffer concentration corresponds to less than 20% increase in rate constants, and demonstrates that at 0.5 mM concentration, the role of the buffer as proton acceptor is negligible.

Table S8: Rate constants obtained from the short component of a double exponential fit to TA traces measured at different buffer concentrations. Samples contained 15 – 25 μM $[ZnTPPS]^{4-}$, 0.4 – 2 mM $[Co(NH_3)_5Cl]^{2+}$ and 0.3 – 0.4 mM NAWEE.

pH (±var)	$[Na_2HPO_4]$ (mM)	k_1/[NAWEE] ($M^{-1}s^{-1}$)
7.8 (±0.2)	0.5	1.2×10^5
7.8 (±0.2)	1	9.3×10^4
7.95 (±0.05)	2.5	1.3×10^5
7.96 (±0.03)	5	1.5×10^5
7.93 (±0.05)	5	1.4×10^5

IV. Excluding OH^- as Primary Proton Acceptor

i. Concerted PCET

For OH^- to act as a proton acceptor, it must be present at sufficient concentration to account for the observed rate constants. Stepwise mechanisms can be excluded; PTET is excluded on the basis of the high pK_a value of the indole proton (~17), and ETPT is excluded on the basis of the rate constants that increase above the value obtained at pH < 4.5 where the mechanism is limited by ET. We therefore only consider the concerted mechanism. For CEPT, we can analyze the reaction as OH^- reacting with the $\{PS_{ox}\cdots W\}$ encounter complex (PS_{ox} = oxidized photosensitizer) after it has formed, see below.

$$PS_{ox} + W \underset{k_{-d}}{\overset{k_d}{\rightleftharpoons}} \{PS_{ox}\cdots W\} \quad (S4)$$

$$\{PS_{ox}\cdots W\} + OH^- \xrightarrow{k_{OH^-}} PS + W^\bullet + H_2O \quad (S5)$$

k_{OH^-} in reaction S5 has a sufficient driving force that it can be treated as an irreversible step, albeit slow compared to the pre-equilibrium in S4 because of the low $[OH^-]$. By applying a steady state approximation on the encounter complex, we get the following expression for the rate:

$$\text{rate} = \frac{d[W^\bullet]}{dt} = k'_{obs}[PS_{ox}][W] \quad (S6)$$

where the pseudo first order rate constant k'_{obs} is defined as:

$$k'_{obs} = \frac{k_d k_{OH^-}[OH^-]}{k_{-d} + k_{OH^-}[OH^-]} \quad (S7)$$

Assuming that there is no specific driving force for formation of the complex in reaction S4, i.e., $\Delta G° = 0$ we get $k_d/k_{-d} = 1\ M^{-1}$. Given that $k_d \approx 10^{10}\ M^{-1}\ s^{-1}$ in water, this allows us to estimate k_{-d} to $\approx 10^{10}\ s^{-1}$. In solution, k_{OH^-} cannot be greater than the rate of diffusion, $k_{diff} \approx 10^{10}\ M^{-1}\ s^{-1}$ in water, and is in fact likely lower. Comparing k_{-d} to $k_{OH^-}[OH^-]$ would even at pH = 10 yield $k_{OH^-}[OH^-] < 10^6\ s^{-1} << k_{-d}$. This assumption allows us to simplify Eq. S7 to Eq. S8, below.

$$k'_{obs} = \frac{k_d k_{OH^-}[OH^-]}{k_{-d}} \quad (S8)$$

Recalling that $k_d/k_{-d} = 1\ M^{-1}$, Eq. S8 can be further simplified to $k'_{obs} = k_{OH^-}[OH^-]/M$ (k'_{obs} is a second-order rate constant) which makes comparison with the experimentally observed k_{PCET} possible and should give $k_{PCET} = k_{OH^-}[OH^-]$ if OH^- is the primary proton acceptor. As k_{OH^-} is $\leq k_{diff}$ ($\approx 10^{10}\ M^{-1}\ s^{-1}$), $[OH^-]$ is not sufficiently large even at pH = 11.4 to yield rate constants that are similar to what was experimentally observed ($k_{PCET} = 3.5\times10^8\ M^{-1}s^{-1}$ at pH = 11.4) with WEE and

$[Ru(dmb)_3]^{3+}$ as oxidant. For NAWEE, the same is found for pH ≥ 9. Thus, we conclude that OH^- cannot be the primary proton acceptor in the pH range examined.

ii. Stepwise PTET

For a PT limited PTET reaction the derivation of k_{PTET} is analogous to the one for k'_{obs}. The rate constant for PT decreases by one order of magnitude for each unit of ΔpK_a. The deprotonation of W with OH^- as proton acceptor have a $\Delta pK_a = -3$. k_{PTET} can therefore be maximum 10^7 $M^{-1}s^{-1}$, which is not enough to account for the observed rate constants for neither WEE nor NAWEE. With the same logic, PT limited PTET with water as proton acceptor ($\Delta pK_a = -17$) can also be ruled out.

REFERENCES

1. Harriman, A., Further comments on the redox potentials of tryptophan and tyrosine. *J. Phys. Chem.* **1987,** *91* (24), 6102-6104.
2. Costentin, C.; Louault, C.; Robert, M.; Saveant, J. M., The electrochemical approach to concerted proton–electron transfers in the oxidation of phenols in water. *Proc. Natl. Acad. Sci.* **2009,** *106* (43), 18143-8.
3. O'Reilly, J. E., Oxidation-reduction potential of the ferro-ferricyanide system in buffer solutions. *Biochim. Biophys. Acta. Bioenerg.* **1973,** *292* (3), 509-515.
4. Zhang, M.-T.; Nilsson, J.; Hammarström, L., Bimolecular proton-coupled electron transfer from tryptophan with water as the proton acceptor. *Energy Environ. Sci.* **2012,** *5* (7).
5. Ener, M. E. Electron Flow Through Cytochrome P450. PhD thesis, California Institute of Technology, 2014.
6. Bonin, J.; Costentin, C.; Robert, M.; Routier, M.; Saveant, J. M., Proton-coupled electron transfers: pH-dependent driving forces? Fundamentals and artifacts. *J. Am. Chem. Soc.* **2013,** *135* (38), 14359-66.
7. Dongare, P.; Maji, S.; Hammarstrom, L., Direct Evidence of a Tryptophan Analogue Radical Formed in a Concerted Electron-Proton Transfer Reaction in Water. *J. Am. Chem. Soc.* **2016,** *138* (7), 2194-9.
8. Sjodin, M.; Styring, S.; Wolpher, H.; Xu, Y.; Sun, L.; Hammarstrom, L., Switching the redox mechanism: models for proton-coupled electron transfer from tyrosine and tryptophan. *J. Am. Chem. Soc.* **2005,** *127* (11), 3855-63.
9. Kerzig, C.; Goez, M., Generating hydrated electrons through photoredox catalysis with 9-anthrolate. *Phys. Chem. Chem. Phys.* **2015,** *17* (21), 13829-36.
10. Lomoth, R.; Häupl, T.; Johansson, O.; Hammarström, L., Redox-Switchable Direction of Photoinduced Electron Transfer in an Ru(bpy)32+-Viologen Dyad. *Chemistry - A European Journal* **2002,** *8* (1), 102-110.
11. Solar, S.; Getoff, N.; Surdhar, P. S.; Armstrong, D. A.; Singh, A., Oxidation of tryptophan and N-methylindole by N3. cntdot., Br2.-, and (SCN) 2.-radicals in light-and heavy-water solutions: a pulse radiolysis study. *J. Phys. Chem.* **1991,** *95* (9), 3639-3643.
12. Tarnovsky, A. N.; Gawelda, W.; Johnson, M.; Bressler, C.; Chergui, M., Photexcitation of aqueous ruthenium(II)-tris-(2,2'-bipyridine) with high-intensity femtosecond laser pulses. *J. Phys. Chem. B* **2006,** *110* (51), 26497-505.

13. Glover, S. D.; Tyburski, R.; Liang, L.; Tommos, C.; Hammarstrom, L., Pourbaix Diagram, Proton-Coupled Electron Transfer, and Decay Kinetics of a Protein Tryptophan Radical: Comparing the Redox Properties of W32• and Y32• Generated Inside the Structurally Characterized alpha3W and alpha3Y Proteins. *J. Am. Chem. Soc.* **2018,** *140* (1), 185-192.
